# Predicting COVID-19 exposure risk perception using machine learning

**DOI:** 10.1186/s12889-023-16236-z

**Published:** 2023-07-18

**Authors:** Nan Zou Bakkeli

**Affiliations:** Centre for Research on Pandemics & Society; Consumption Research Norway, Oslo Metropolitan University, P.O. Box 4, St Olavs Plass, Oslo, 0130 Norway

**Keywords:** Exposure risks, Risk perception, COVID-19, Health inequality, Social determinants of health, Occupational health, Interpretable machine learning

## Abstract

**Background:**

Self-perceived exposure risk determines the likelihood of COVID-19 preventive measure compliance to a large extent and is among the most important predictors of mental health problems. Therefore, there is a need to systematically identify important predictors of such risks. This study aims to provide insight into forecasting and understanding risk perceptions and help to adjust interventions that target various social groups in different pandemic phases.

**Methods:**

This study was based on survey data collected from 5001 Norwegians in 2020 and 2021. Interpretable machine learning algorithms were used to predict perceived exposure risks. To detect the most important predictors, the models with best performance were chosen based on predictive errors and explained variances. Shapley additive values were used to examine individual heterogeneities, interpret feature impact and check interactions between the key predictors.

**Results:**

Gradient boosting machine exhibited the best model performance in this study (2020: RMSE=.93, MAE=.74, RSQ=.22; 2021: RMSE=.99, MAE=.77, RSQ=.12). The most influential predictors of perceived exposure risk were compliance with interventions, work-life conflict, age and gender. In 2020, work and occupation played a dominant role in predicting perceived risks whereas, in 2021, living and behavioural factors were among the most important predictors. Findings show large individual heterogeneities in feature importance based on people’s sociodemographic backgrounds, work and living situations.

**Conclusion:**

The findings provide insight into forecasting risk groups and contribute to the early detection of vulnerable people during the pandemic. This is useful for policymakers and stakeholders in developing timely interventions targeting different social groups. Future policies and interventions should be adapted to the needs of people with various life situations.

**Supplementary Information:**

The online version contains supplementary material available at 10.1186/s12889-023-16236-z.

## Introduction

The COVID-19 pandemic has posed considerable challenges in people’s daily lives. Although pandemics are random in nature, each individual’s vulnerability to exposure is unequally distributed. Studies have found that people with lower socioeconomic status, precarious employment and poor living conditions are more exposed and face larger health challenges than others [1–3]. Exposure to COVID-19 not only impacts people’s physical health, but is also a risk factor for mental health problems [4, 5]. Studies have identified exposure risk as the most important predictor of mental illness and diminished life satisfaction [6–9]. Although previous studies have examined correlations or causal relationships between exposure risk and factors like socioeconomic status, demographic background, psychological factors and epidemiological and pathological determinants [1, 10–12], more studies are needed to predict the outcome of exposure risks. It is also crucial to systematically explore the most important determinants of exposure risk in different pandemic phases.

Exposure risk has been defined in various ways in COVID-19 studies. The term is often associated with the estimation of disease probability for certain demographic groups [13, 14] or psychological measures of fear or cognitive process [15, 16]. However, exposure can both be determined by external structural impact and reflect on individuals’ perceptions of the situation they are in. A sociological perspective of self-perceived exposure risk can thus contribute to understanding vulnerability by combining individual agency with structural environment, social position and contextual situation. There is, therefore, a need to conduct more sociological studies on exposure risk, explain why some factors predict exposure risk more strongly, and interpret how predictions vary among individuals and social groups.

The present study examines self-perceived exposure risk and predicts level of exposure by using supervised machine learning. The predictors consist of a wide range of features that give rich information on demographics, socioeconomic status, work and occupation, social contact, COVID-related health condition and risks, and social epidemiological, intentional and behavioural factors. Using data from 2020 and 2021, we (1) detect important contributors to exposure, (2) interpret the overall effect and importance of the top-ranked variables and (3) examine individual variability and heterogeneity when predicting exposure risk.

This study aims to contribute to the existing literature in several ways. First, instead of looking at the *relationships* between certain factors and vulnerability, the study contributes to *predicting* the degree of exposure. By including numerous predictors, this study incorporates complex social realities into the predictive models. Second, instead of limiting the focus to psychometrics of fear or estimations of morbidity, this study offers a broader definition of exposure. Psychological distress, fear and emotions are closely related to both the ‘hard fact’ of morbidity risks and an individual’s opportunities and limitations grounded in structural settings. Incorporating rich information about social, behavioural and epidemiological aspects, this study provides a comprehensive view of exposure during the pandemic. Furthermore, we aim to open up the machine learning ‘black box’ using interpretability models. By visualising individual variability in prediction models, this study can both contribute to increased algorithmic transparency and take individual heterogeneity into account. Capturing variations among different individuals and social groups, the results can be useful for adjusting interventions targeting various social groups in different pandemic phases.

## Theories and previous studies

### Sociology of exposure risk

The concept of exposure risk in COVID-19 varies across research fields. In medical studies, the term has been used interchangeably with morbidity or mortality risk, which measures the probabilities of disease transmission for different populations and groups [13, 14]. In psychology, a large body of literature relates exposure risk to fear, cognitive processes, self-efficacy and emotional responses to disasters [15, 17]. The disciplinary field of risk assessment often looks at risk as a process to identify potential hazards and detect exposure to an uncertain proposition, such as chemical exposure or environmental hazards [18, 19].

A sociological perspective of risk takes into consideration individual perceptions of experiences in combination with contextual and structural understanding of exposure risks. Beck (1992) argued that concerns about danger and hazards were central in contemporary society [20]. A risk society implies fading borders of uncontrollable risks, and this border fading has become highly visible in the COVID-19 pandemic [21]. For sociological understanding, risk perceptions are not purely a result of cognitive processes, but are also intertwined with cultural and social structures [22]. Risks are objective and real, but people’s responses to risk are based on a common understanding of the objective situation in combination with their own social position and life situation. The concept of exposure risk is therefore related to the way in which people perceive themselves, affected by expert definitions and their own experience and perception [22].

For example, if in an ‘at risk’ group, individuals may feel the need to reassure certainty, as well as seek out more interventions to protect themselves from the risk [22]. During the pandemic, medical and public health experts suggest that certain groups are at a higher exposure risk for the coronavirus. This could potentially influence people who belong to certain sociodemographic groups to alter their behaviour and re-evaluate their exposure.

Moreover, risk perception interacts with one’s social position and contextual factors [23]. Individual evaluation of exposure risks can largely depend on one’s life situation, and the ability to comply with recommendations. More concretely, work, livelihood and family obligations can all determine how much a person is able to keep social distancing, is exposed to the disease, and feels more or less safe in different contextual situations [1, 24, 25]. Therefore, self-perceived exposure risk is not only a matter of the clinical probability of virus transmission or cognitive heuristics, but also depends on people’s socioeconomic positions and their experience of being situated in different social arenas in everyday life.

In this study, we explore and predict individuals’ self-perceived exposure risk for COVID-19, based on demographic, socioeconomic, epidemiological and behavioural factors. Instead of explicitly focusing on the aspect of fear or the clinically estimated probability of getting sick, we look at self-perceived risk as a concept that captures some psychological and epidemiological aspects of the pandemic impact and adds social and contextual elements into an individual’s interpretation of their own situation. We use ‘exposure risk’ and ‘exposure’ interchangeably.

### Social inequality in vulnerability

Vulnerability in exposure risk is inequitably distributed among people with different demographic, social and epidemiological backgrounds. For example, in the U.S., women have reported more worry about COVID-19 exposure, along with ethnic minorities and older groups [26]. Minorities tend to have a higher exposure risk because they often have jobs that involve direct contact with others [27]. In Sweden, fear of exposure is higher for women, those who have had mental health treatment, those perceived to be at risk and people who are not working or studying [28].

Lower socioeconomic status is associated with an increased risk of mortality [29–31]. Economically disadvantaged people tend to have poorer housing conditions with limited access to personal outdoor space, are more likely to be employed in occupations with a higher risk of exposure, and live with higher stress, which weakens the immune system [32]. Vulnerability to exposure risk is highly related to socioeconomic status. People with lower status have less access to daily necessities, protective supplies and information that those with higher socioeconomic status; they are less likely to retain their jobs or work remotely and often live in communities with frequent close contact with others [1].

Occupational exposure is a key factor in exposure risk in the pandemic. Sandal and Yildiz (2021) stated that the pandemic ‘can be categorized as an occupational disease, because employees, particularly in the healthcare system, can be infected at the workplace’ [33]. Exposure to COVID-19 disproportionately affects specific occupations and industries in which essential workers are more likely to be exposed [34]. In the U.S., a large proportion of foreign-born ethnic groups work in high-exposure-risk occupations [35]. Based on health data from 356,188 working-age individuals, Chen et al. (2021) found that Latinos in the food or agriculture sector, black people in the transportation or logistics sector, and white people in manufacturing facilities had the highest mortality rates [36]. The prevalence of fear of exposure or transmission was higher among nursing assistants and black and Latino workers than white workers and higher among women than men [37]. Khunti et al. (2020) called attention for the fact that ethnic minority groups have been disproportionately affected by COVID-19 [38]. For people with ethnic origins, worry about exposure may be caused by structural racism and lower socioeconomic status, including housing quality, economic situations and healthcare opportunities [39].

### Fear and emotional response

Emotional responses to pandemics, such as fear of being exposed to the coronavirus or worry about the present situation, are instrumental for people’s behaviour and daily praxis. Scholars found that protective behaviour during COVID-19 was not influenced by political orientation or moral perceptions, but was more often caused by fear [15]. Previous studies have identified fear as the most important factor predicting key attitudes and individual virus-mitigating behaviour in the context of COVID-19 [15, 28].

Exposure risk has a strong negative impact on risk taking [40]. Individuals who perceive themselves to be more at risk, such those with higher health anxiety, are more likely to report fear [41] and follow protective measures in epidemics [42]. Some other studies have focused on the role of self-efficacy in behavioural measures in the pandemic. Self-efficacy is the degree to which a person masters knowledge about COVID-19 and to what extent they follow recommendations. Experiencing fear about a threat or crisis can cause behavioural and attitudinal change [43]. In Western democratic countries, self-efficacy decreases the perceived relevance of threat in a large-scale and immediate crisis such as the pandemic, and thus contributes more to individual behaviour than fear [17].

Risk perception in psychology refers to either logical risk analysis and decision making or an individual’s emotional and heuristic response [44]. Heuristic psychology emphasises the (in)consistency between self-assessed risk and risk adjustment. Slovic et al. (1980) argued that when evaluating risks, people rely on inferences based on their memories [45]. Cognitive limitations, combined with aversion to hazards and biased media coverage, can mislead people’s perceptions. However, this approach moves away from individual agency and structure. Individuals’ actions and decisions often reflect structural impact, and exposure to COVID-19-related risk is closely related to people’s opportunities, social positions and options [1, 27].

In this study, we look at self-perceived exposure, which may involve a complex interplay between sociopsychological and epidemiological factors. We examine the level of exposure with a variety of social, epidemiological and behavioural conditions. There can be, for example, large heterogeneities in self-perceived exposure risk among people with the same degree of compliance. While some complied because they had opportunities, others complied due to fear. To handle a large number of predictor variables while explaining complex individual variations, the following section presents the praxis of interpretable methods that contribute to opening up the black box.

## Interpreting the ‘black box’

Machine learning methods have become increasingly useful for predictive studies, as well as for the social sciences. Machine learning algorithms are advantageous in predictive studies because they effectively capture generalisable predictive patterns in the data set. They are flexible, explorative and able to handle multidimensional and multi-variety data in dynamic environments. Furthermore, some machine learning models, such as ensemble methods, have been shown to outperform traditional linear regressions with higher model accuracy and fewer errors.

However, machine learning algorithms have been criticised as black boxes that lack transparency and are difficult to interpret. Traditionally, simple models like linear regression are preferred for their transparency and interpretability. Nevertheless, such models suffer from the problem of overfitting, are not able to include many predictors, and face the challenge of lower prediction performance.

Model interpretability and explainability have become important topics in recent years. The trade-off between model performance and interpretability has become a critical hindrance for the more widespread use of machine learning techniques, especially in policy making. In high-stakes domains, such as the field of medicine and healthcare, stakeholders and professionals tend to prefer more traditional interpretable prediction models over machine learning [46], even though the latter often provides better results. Therefore, it is crucial to develop studies that can unify the high prediction performance and apply algorithms that provide a more intuitive understanding of the results produced.

New approaches have been changing the trends, combining global and local interpretations of machine learning models and visualising the results in a more transparent way. One approach to developing more interpretable machine learning analysis is to describe what the model has learned and to explore the research questions using stable information extracted from the best-fitted models. Such information can be displayed by anticipating domain relationships in the form of scatter plots or histograms [47].

For example, Datta et al. (2016) explored the likelihood of arrest and designed an algorithmic analysis of fairness to verify that the algorithm was not discriminating against people based on protected attributes, such as race or gender [48]. By showing transparency reports for a single individual (‘Mr. X’), scholars were able to measure the influence of inputs on decisions made by individuals versus groups. Using Shapley values based on game theory, identified key players in terrorist networks, incorporating both the structure of the terrorist network and individual factors, such as financial means or bomb-building skills [49]. Based on individual conditional expectation curves, Lee and Lee (2022) illustrated the complex relationships between transit ridership and individual factors in urban areas in the United States, and found that the key determinants of decreased public transportation use included a combination of decreases in carless households and gasoline prices [50]. Such studies have also been carried out for COVID-19. Study identified key features that contributed to predictions of true claims and false news about COVID-19 using complex text data [51]. Analysing situational information on social media about various disasters, Behl et al. (2021) provided model explanations that verified model behaviour by looking at individual instances, based on local, interpretable model-agnostic explanations [52]. By applying interpretability methods, such studies are able to embrace complex social realities and interactions in predictive analysis.

In this paper, we adopt interpretability machine learning models to predict exposure risk using demographic, socioeconomic, behavioural and epidemiological factors. We further explore the important predictors and explain how they contribute to the outcome of exposure risk. We also examine individual variations. The analysis integrates individual-level interpretation with global explanations of the general trend, providing more detailed and transparent descriptions of the black box method.

## Methods

### Data and variables

We used the CorLife survey data from Consumption Research Norway and the Work Research Institute. The survey contained responses from 5,001 individuals aged 18–80 years during 2020 (3,001 respondents) and 2021 (2,000 respondents). The baseline data ($t_{1}$) was collected from 1,000 respondents from 18–23 March 2020, when the COVID-19 pandemic first hit Norway. The second wave of data ($t_{2}$) was collected from 2,001 respondents from 13–22 May 2020, when Norway recorded the first peak of the pandemic. One year later, during Norway’s second peak, a third wave ($t_{3}$) was collected from 2,000 respondents from 21 April to 5 May 2021.

The target variable was *self-perceived exposure risk*. We employed self-assessments to investigate exposure to the coronavirus disease in four arenas. The respondents were asked, ‘How exposed to COVID-19 are you at home/in neighbourhood/at store/at work?’. The respondents chose a number from 1 (‘mostly exposed’) to 5 (‘not exposed’) for each arena. Cronbach’s alpha for the four exposure variables was .78 in 2020 and .74 in 2021. An indicator was then constructed using principal component analysis. The final indicator subtracted the lowest value to provide a more intuitive interpretation. The variable of exposure risk ranged from 0 to 6.89, where a higher value indicated an increased level of self-evaluated exposure risk ($\mu _{2020}=2.05, \sigma _{2020}=1.03$; $\mu _{2021}=1.51, \sigma _{2021}=1.09$).

The study included 54 features to predict exposure risk. *Socioeconomic and demographic features* included age, gender, geographic residence, education, income and household types. *Work and occupational features* included employment status, occupation, work in essential sectors and work-life conflict. *Behavioural features* consisted mobility pattern, social distancing, willingness to stay at home, social contact and compliance with nonpharmaceutical interventions (NPI). *Epidemiological features* were measured by COVID-19-related disease symptoms and risk factors. See Additional file 1: Appendix B and C for detailed information about data and variables.

### Analytical procedure

We deployed supervised learning to predict the degree of self-perceived exposure risks in 2020 and 2021, separately for each year. The wave 1 ($t_{1}$) and 2 ($t_{2}$) surveys were merged to analyse situations in 2020, while the wave 3 ($t_{3}$) survey was used for the 2021 analysis. For each of the 2020 and 2021 datasets, we cleaned the dataset and imputed missing values before the datasets were divided into training (60%) and test sets (40%). The training set was used to tune the hyperparameters in the models while the test set was used to predict self-perceived exposure risks.

We applied a grid search to optimise the models. When a hyperparameter space was specified, we repeated the 10-fold cross-validation ten times in the training set to plot the learning and training curves. The final models were run with the test set, where we used root mean squared errors (RMSE), mean absolute errors (MAE) and the coefficient of determination (RSQ) to evaluate model performance.

**Figure:**
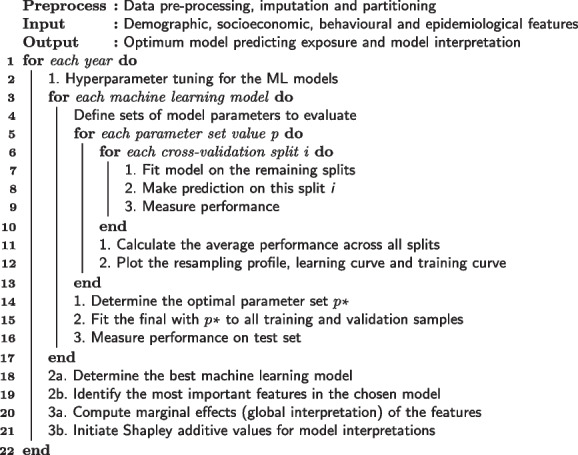
**Algorithm 1** Workflow in machine learning

We chose the best models based on their performance to identify important features in predicting self-perceived exposure risk, show the marginal effect of the most important features on the predicted outcome of the models and assess model explanations of the features. See Algorithm 1 for detailed information on model tuning and the analytical procedure of this study.

### Machine learning models

We optimised four machine learning algorithms that represent a broad approach to predicting self-perceived exposure risks: gradient boosting machines (GBM), elastic net regularisations (ENR), support vector machines (SVM) and k-nearest neighbour (KNN).

*GBMs* are based on ensemble techniques. Starting with fitting an initial tree model for each iteration, a new model is added to the ensemble sequentially and trained with respect to the error of the previous model. Therefore, each case is based on the gradient of the error—the combination of individual models creates a more powerful new prediction that minimises the overall prediction error. More specifically, given the loss-function $\Psi (y,f)$ and the base-learner model $h(x,\theta )$, the negative gradient $g_t(x)$ will be computed to fit a new base-learner function $h(x,\theta _t)$. The best gradient descent step-size $\rho _t$ can be computed by $\rho _t = \arg \min _\rho \sum _{i=1}^N\Psi [y_i,\widehat{f_{t-1}}(x_i)+\rho h(x_i,\theta _t)]$, where $\hat{f_0}$ is initialised with a constant [53].

*ENRs* are penalised linear regressions with a penalty term added to the mean squared errors. The loss function penalises large model parameters and reduces overfitting. This technique combines ridge and lasso regularisations: $\lambda (\frac{1}{2} (1-\alpha )\sum _{j=1}^p\beta _{j}^2+\alpha \sum _{j=1}^p|\beta _{j}|)$, in which the penalty parameter $\lambda$ controls the amount of shrinkage, while the parameter $\alpha$ controls the type of shrinkage [54].

*SVMs* are supervised learning models that find the optimal hyperplanes (i.e. the simplest decision boundary) for classification and regression [55]. The SVM algorithm chooses a hyperplane from which the distance between the hyperplane and the nearest data points is optimal. To identify the best boundary, the SVM kernel transforms low-dimensional input space into a higher-dimensional feature space. The radial basis kernel function calculates the exponential of the squared Euclidean distance between two data points, $\textbf{x}$ and $\mathbf {x'}$: $K(\textbf{x},\mathbf {x'}) = \exp (-\frac{\Vert \mathbf {x-x'}\Vert ^{2}}{2\sigma ^{2}})$. A higher value of the parameter $\gamma =1/2\sigma ^{2}$ gives a clearer decision boundary between data points.

Finally, *KNN* assumes that similar cases exist in proximity and captures similarities by calculating the distance between observations. The KNN regression uses the neighbouring observations of the new observation to suggest the target outcome. The algorithm estimates the conditional distribution of the target variable given a matrix of features and predicts an observation with the highest probability: $\hat{f_k}(x) = \frac{1}{k}\sum _{i \in \mathcal {N}_k(x,\mathcal {D})} y_i$. Here $\mathcal {N}_k(x,\mathcal {D})$ denotes the set of *k*-nearest observations, and the distance function is based on the Minkowski distance $\textrm{d}(\textbf{x},\textbf{z})=(\sum _{r=1}^d |x_r-z_r|^p)^{1/p}$ (see, e.g., [56]).

### Model interpretation

After selecting the models exhibiting desirable performance, we identified the most important features in predicting self-perceived exposure risks. With the chosen variables, we first provided a global interpretation of the model, quantifying the overall relationship between the target and the features. Next, we examined how individual instances vary for the features of interest by showing the individual conditional expectation (ICE) curves. The ICE plots visualise how each individual prediction changes with the feature value [57]. In this study, the ICE curves were centred to display the predictive differences related to the centred point: $\hat{f}_{cent}^{(i)}=\hat{f}_{}^{(i)}-\textbf{1}\hat{f}(x^{a},x_C^{(i)})$, where $\hat{f}$ is the fitted model, $x^{a}$ is the anchor point, and the vector $\textbf{1}$ denotes number of dimensions [58].

Finally, we explained the model’s constituent features by calculating the Shapley additive values (SHAP). Using additive feature attribution methods, the SHAP value is the average marginal effect of a feature across all possible combinations [58, 59]. These values measure the contribution to the final model outcome from each individual observations separately, and the total change in the prediction of the model is divided among the features with respect to their contributions across all possible subsets of features. The amount a feature *i* is given in a coalition (*v*, *N*) is calculated by: $\phi _{i}(v)=\sum _{C\subseteq N-i}\frac{|C|!(n-|C|-1)!}{n!}\lbrace v(C\cup \lbrace i\rbrace )-v(C)\rbrace$, where $N-i$ denotes the set *N*, and $|C|$ is the cardinality of *C*. The weight factor $\frac{|C|!(n-|C|-1)!}{n!}$ is the probability that the number of *C* are ahead of a specific feature *i* in any permutations, and the term $v(C\cup \lbrace i\rbrace )-v(C)$ gives the marginal contributions of feature *i* if *i* joins the coalition after all features in *C* have joined.

### Robustness tests

Comparing GBM, ENR, SVM and KNN can enable the assessment of the general performance of model predictions. We have performed several additional robustness tests to interpret the models. First, we provided a global interpretation to illustrate the overall relationship between exposure risk and the most important features by estimating the marginal effects of the features on the predicted outcomes, which were plotted using partial dependence plots (PDPs), as presented in Additional file 1: Appendix D. PDPs are based on the average predicted outcome when holding other features constant; they may be simplified and may obscure potentially interesting relationships between individual instances. Therefore, the combination of PDPs and ICE curves is ideal to capture both global trends and individual variations.

Second, we have uncovered local variations using local interpretable model-agnostic explanations (LIME) as an additional test (Additional file 1: Appendix F). One way of assessing local explanations is to zoom into single cases. In this analysis, we show some randomly selected cases (individuals) in each year; their most important explanatory features in relation to exposure risk; and how each of the features contribute for each individual.

## Results

### Descriptive statistics

Before running the machine learning models, we show the descriptive statistics for the attributes of all the predictor variables and the target variable in the original datasets (before data partitioning). See Table 1.

**Table 1:** Descriptive statistics

	Self-perceived exposure risk (2020)			Self-perceived exposure risk (2021)		
	Mean (SD)	N $(\mu _y)^{\bigstar }$	Coef. (SE)^✤^	Mean (SD)	N $(\mu _y)^{\bigstar }$	Coef. (SE)^✤^
**Demographic and socioeconomic factors**						
*Age*						
18-22	.10 (.30)	292 (2.18)	Ref.	.11 (.32)	229 (1.84)	Ref.
23-35	.25 (.43)	734 (2.35)	.18(.07)**	.24 (.43)	473 (1.76)	-.08(.09)
36-55	.33 (.47)	980 (2.00)	-.18(.07)**	.33(.47)	649 (1.65)	-.19(.08)*
56-80	.33 (.47)	969 (1.50)	-.68(.07)***	.32(.47)	640 (1.38)	-.47(.08)***
*Gender*						
Male	.46 (.50)	1,370 (1.74)	Ref.	.46(.50)	923 (1.45)	Ref.
Female	.53 (.50)	1,605 (2.13)	.39(.04)***	.54 (.50)	1,068 (1.75)	.29(.05)***
*Education*						
High	.40 (.50)	1,202 (1.92)	Ref.	.35(.48)	699 (1.60)	Ref.
Low	.59 (.50)	1,773 (1.98)	.06(.04)	.64(.48)	1,292 (1.64)	-.04(.05)
*Income*	3.31 (1.35)	2,975 (1.95)	1.96(.05)***	3.46 (1.38)	1,991 (1.61)	-.01(.02)
*Geography*						
Oslo	.15 (.36)	458 (2.16)	Ref.	.15(.36)	300 (1.85)	Ref.
>50,000	.29 (.45)	868 (2.02)	-.13(.06)*	.30 (.46)	605 (1.60)	-.25(.08)**
5,000-50,000	.29 (.45)	853 (1.95)	-.20(.06)**	.31 (.46)	610 (1.64)	-.22(.08)**
2,000-4,999	.13 (.33)	383 (1.84)	-.31(.07)***	.11 (.32)	224 (1.60)	-.26(.10)**
<2,000	.14 (.35)	413 (1.63)	-.53(.07)***	.13 (.33)	252 (1.30)	-.55(.09)***
*Household type*						
Couple & child.	.25 (.43)	748 (2.07)	Ref.	.23 (.42)	461 (1.77)	Ref.
Couple no child.	.35 (.48)	1,042 (1.86)	-.21(.05)***	.35 (.48)	691 (1.50)	-.27(.06)***
Living alone	.06 (.24)	181 (2.09)	.02(.09)	.06 (.24)	120 (1.60)	-.17(.11)
Single parent	.24 (.43)	725 (1.85)	-.22(.05)***	.24 (.43)	477 (1.51)	-.26(.07)***
Other types	.09 (.29)	279 (2.07)	-.00(.07)	.12 (.33)	242 (1.82)	.05(.09)
**Work and occupation**						
*Currently employed*						
No	.49 (.50)	1,466 (2.10)	Ref.	.51 (.50)	912 (1.65)	Ref.
Yes	.51 (.50)	1,509 (1.78)	.32(.04)***	.46 (.50)	1,079 (1.58)	-.07(.05)
*Occupation*						
Leaders	.04 (.19)	106 (1.81)	Ref.	.03 (.17)	57 (1.62)	Ref.
Professionals	.10 (.29)	283 (1.99)	.18(.11)	.11 (.31)	214 (1.54)	-.08(.16)
Technicians	.10 (.29)	286 (2.08)	.26(.11)*	.07 (.26)	140 (1.79)	.16(.17)
Clerical support	.09 (.27)	243 (1.99)	.18(.11)	.10 (.30)	197 (1.47)	-.16(.16)
Service & sale	.13 (.33)	381 (2.36)	.55(.11)***	.10 (.30)	205 (1.67)	.04(.16)
Agriculture	.01 (.08)	18 (1.40)	-.41(.25)^†^	.01 (.07)	11 (1.35)	-.28(.36)
Craft & trades	.03 (.17)	90 (2.10)	.29(.14)*	.03 (.16)	55 (1.54)	-.09(.20)
Plant & machine	.02 (.14)	62 (2.07)	.26(.16)	.02 (.13)	35 (1.63)	.01(.23)
Elementary	.02 (.13)	49 (2.19)	.38(.17)*	.03 (.16)	51 (1.69)	.07(.21)
Job-seeker	.13 (.33)	375 (2.03)	.22(.11)*	.14 (.35)	288 (1.79)	.17(.16)
Retirees	.17 (.37)	496 (1.32)	-.49(.11)***	.16 (.36)	314 (1.38)	-.25(.16)
Sickness leave	.01 (.12)	42 (2.14)	.32(.18)^†^	.01 (.08)	14 (2.06)	.43(.32)
Disability benefit	.07 (.25)	203 (1.77)	-.05(.12)	.07 (.26)	149 (1.67)	.05(.17)
Other	.11 (.32)	341 (2.16)	.35(.11)**	.13 (.34)	261 (1.67)	.05(.14)
*Essential worker*						
No	.82 (.38)	2,450 (1.87)	Ref.	.17 (.37)	1,660 (1.59)	Ref.
Yes	.18 (.38)	525 (2.30)	.42(.05)***	.83 (.37)	331 (1.72)	.13(.07)*
**Work-life conflict**	1.67 (.75)	2,975 (1.95)	.15(.03)***	1.62 (.73)	1,991 (1.61)	.20(.03)***
**Behavioural and preferance predictors**						
*Number of ...*						
...times outdoor	2.84 (1.03)	2,975 (1.95)	-.06(.02)**	3.09 (.99)	1,991 (1.61)	-.08(.02)**
...people < 1 m	2.48 (1.31)	2,975 (1.95)	.09(.01)***	2.54 (1.29)	1,991 (1.61)	.02(.02)
*Willingness stayhome*	3.97 (1.10)	2,975 (1.95)	.07(.02)***	3.78 (1.23)	1,991 (1.61)	.08(.02)***
*NPI practice*	4.86 (1.91)	2,975 (1.95)	.12(.01)***	5.49 (2.01)	1,991 (1.61)	.11(.01)***
*Contact with ...*						
...family/friends	3.00 (1.06)	2,975 (1.95)	.14(.02) ***	2.90 (1.10)	1,991 (1.61)	.13(.02)***
...colleagues/work	2.72 (1.32)	2,975 (1.95)	.02(.01)	3.11 (1.40)	1,991 (1.61)	.02(.02)
**Social epidemiological predictors**						
*Transmission risk*						
Risk group (old)	0.29 (0.45)	863 (1.81)	-.19(.04)***	.33 (.47)	657 (1.57)	-.06(.05)
Contacted sick	0.41 (0.49)	1,215 (2.10)	.26(.04)***	.30 (.46)	596 (1.78)	.24(.05)***
Quarantine	0.10 (0.30)	294 (2.06)	.13(.06)*	.17 (.38)	345 (1.91)	.36(.06)***
Isolation	0.04 (0.19)	114 (2.39)	.46(.10)***	.06 (.23)	111 (2.13)	.55(.11)***
Transmitted	0.01 (0.05)	17 (2.55)	.60(.39)	.01 (.10)	19 (1.67)	.06(.25)
Pregnant	0.01 (0.09)	27 (2.61)	.68(.20)**	.01 (.10)	21 (1.78)	.17(.24)
None	0.36 (0.48)	1,065 (1.85)	-.15(.04)***	.34 (.47)	680 (1.46)	-.23(.05)***
*Corona symptom*						
Cough	0.10 (0.30)	294 (2.12)	.19(.06)**	.05 (.22)	97 (1.77)	.17(.11)
Heavy breath	0.06 (0.24)	177 (2.24)	.31(.08)***	.04 (.20)	79 (1.75)	.15(.12)
Fever	0.04 (0.18)	105 (2.15)	.21(.10)*	.01 (.09)	17 (1.74)	.13(.26)
Muscle pain	0.10 (0.29)	280 (2.26)	.35(.06)***	.08 (.26)	150 (2.05)	.48(.09)***
Diarrhoea	0.05 (0.23)	163 (2.33)	.41(.08)***	.03 (.17)	58 (2.08)	.49(.14)**
Tiredness	0.15 (0.36)	443 (2.29)	.41(.05)***	.12 (.33)	243 (2.04)	.48(.07)***
None	0.72 (0.45)	2,146 (1.86)	-.32(.04)***	.80 (.40)	1,593 (1.54)	-.37(.06)***
*Target variable*	Mean (SD)	N	[min,max]	Mean (SD)	N	[min,max]
Self-perceived exposure risk	1.96 (1.03)	2,975	[0,5.24]	1.61(1.09)	1,991	[0,6.89]

Note. *** $p<.001$; ** $p<.01$; * $p<.05$; ^†^ $p<.10$. ${}^{\bigstar }\ \mu _y$ is the mean self-perceived exposure score for the different feature attributes. ^✤^ The estimated coefficients and standard errors are based on bivariate ordinary least squares

In 2020, women, younger people, people with lower education and lower income, Oslo residents, couples with children, essential workers, occupations such as services and sales, technicians, craft and trades and those with elementary jobs, jobseekers, higher work-life conflict and NPI-compliance were correlated with higher perceived risk. Number of people in close proximity, social contacts, willingness to stay at home, transmission risk and all COVID-19-related disease symptoms also increased the perceived exposure risk. The trend in 2021 was very similar to that in 2020. However, household income, occupation, the number of people in close proximity to another and some of the COVID-19 symptoms were no longer significantly correlated with perceived exposure risk.

### Machine learning performances

Table 2 shows a comparison of the machine learning model performances in 2020 and 2021. We measured the model performance by the percentage of explained variance and prediction errors. In both years, the GBM model showed the best performance.

**Table 2:** Prediction performance of self-perceived exposure risk, 2020 and 2021

	2020			2021		
Model/Measure	RMSE	MAE	RSQ	RMSE	MAE	RSQ
Gradient boosting machines (GBM)	0.929	0.735	0.215	0.994	0.774	0.117
Elastic net regularisations (ENR)	0.934	0.738	0.198	1.005	0.786	0.098
Support vector machines (SVM)	0.935	0.733	0.201	1.009	0.775	0.097
K-nearest neighbours (KNN)	0.962	0.759	0.188	1.022	0.787	0.098

The models were optimised to achieve lower prediction errors. When running these models with a selected hyperparameter with the test set, most of them produced an error value of around one. The GBM model had the lowest root mean square error (RMSE) in both years, meaning that the model was able to minimise prediction errors when comparing the observed and predicted outcomes. The RMSE for the GBM model was .93 in 2020 and .99 in 2021. In other words, the deviation between our predictions and the ‘true’ outcome was less than one score of the exposure risk measure. Measured by the mean absolute error, the models with the lowest deviance were SVM in 2020 (.73) and GBM in 2021 (.77).

The model performance measured by the value of RSQ is also fairly satisfying. In social sciences, RSQ values of .02, .13 and .26 can be considered small, medium and large, respectively [60]. In 2020, the GBM explained about 22% of the total variance in self-reported exposure risk, compared to the ENR (20%), SVM (20%) and KNN (19%). In 2021, the GBM model explained 12% of the total variance, compared to the other models with an RSQ value of around 10%. The GBM model has, therefore, achieved both medium-high proportion of explained variance and low errors.

**Fig. 1:**
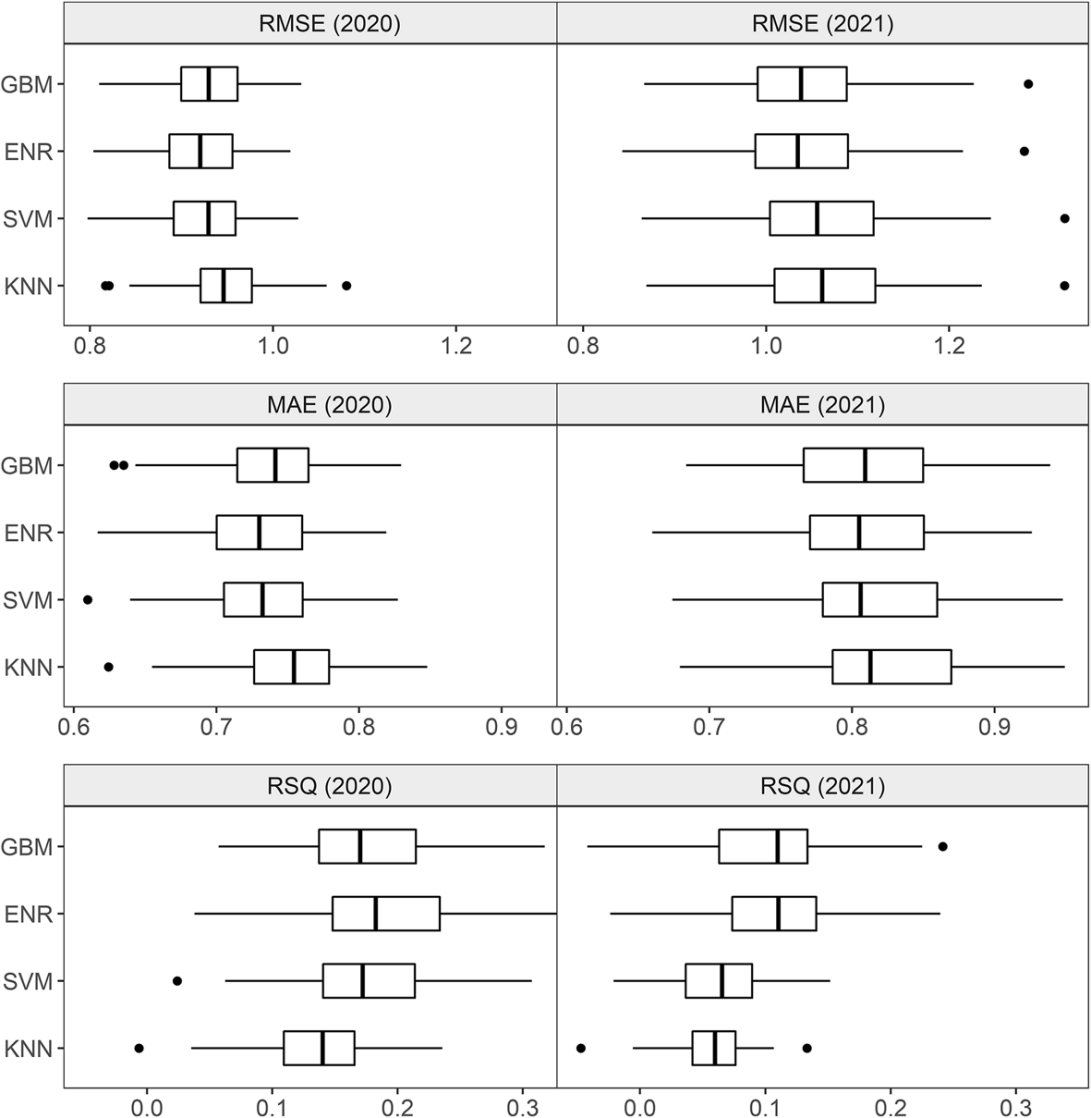
Resampling of accuracies when predicting the test set with the chosen hyperparameters

To verify whether the GBM performs satisfactorily, we resampled the final models with the selected hyperparameter space using the test set to examine variations in the accuracy measures (Fig. 1). The resampling results showed some variations in the accuracy values, which is expected because the sample size was greatly reduced when resampled inside the test set (which was already only 40% of the whole sample size). When comparing the model medians, the GBM still emerged as one of the best models, followed by the elastic-net regularization model.

### Feature importance

Next, we explored how each feature contributes to the predication of exposure risk. We identified the most important variables in 2020 and 2021 using the GBM and ENR models (as a robust check). The calculation of feature importance was based on a permutation method that computed the difference between the baseline performance and the performance obtained after permutation [61]. Since the permutation approach introduces randomness, we performed multiple runs. The values of each feature were randomly permuted 30 times and the average scores were used for importance ranking.

**Fig. 2:**
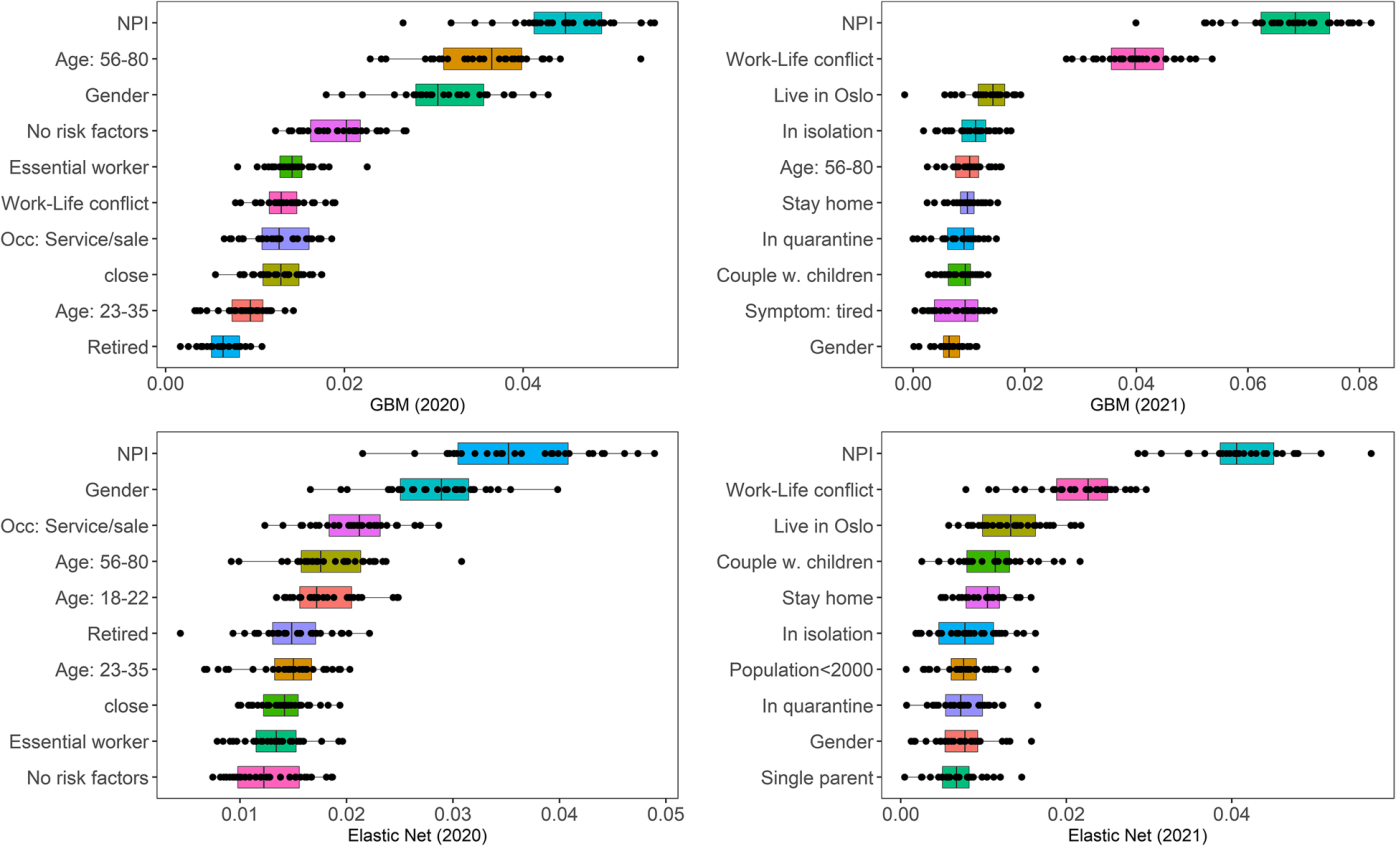
The 10 most important variables in 2020 and 2021

Figure 2 shows the ten most important variables in the two years. The points in the plots illustrate the results of each of the 30 runs. There were small differences in the ranking order when comparing the GBM and ENR models; however, both identified the same important factors for predicting exposure to a large extent. The primary trend, based on both the models, shows that age, gender, the extent of compliance to NPI recommendations and the level of work-life conflict were central in predicting perceived risks in both the years. Zooming into each year, we observed that work-related factors (working in services and sales, being an essential worker and having a retired status), social distancing and susceptibility to COVID-19-related risks played a central role in 2020. In 2021, factors related to daily life (e.g. residential location, homestaying, household structure) and COVID-19-related epidemiological conditions (being in quarantine or in isolation) were more important in predicting self-perceived exposure risk.

### Heterogeneity among individuals

Based on the GBM and ENR models, generally, a higher level of NPI, work-life conflict, being female, being older (56–80 years), living in Oslo and willingness to stay at home correlated with higher predicted exposure risk. See partial dependence plots in Additional file 1: Appendix D for the overall relationship between perceived exposure and the feature variables. Some correlations, such as the positive association between greater perceived exposure risk and a higher level of compliance to NPI and willingness to stay at home, seem counter-intuitive. Explaining the model by evaluating the overall drivers of its predictions across all individuals does not provide a complete picture; there can also be variations among individuals.

**Fig. 3:**
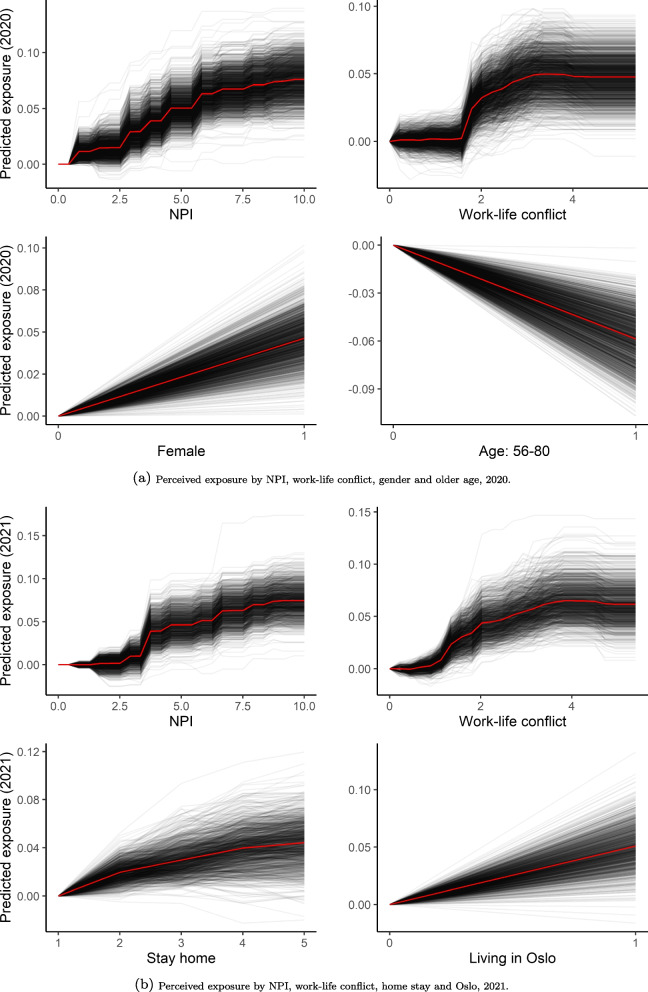
ICE curves for predicting self-perceived exposure risk. **a** Perceived exposure by NPI, work-life conflict, gender and older age, 2020. **b** Perceived exposure by NPI, work-life conflict, home stay and Oslo, 2021

We illustrated this by summarising the individual conditional expectations (ICE) for four important features selected from each year (Fig. 3). There were considerable variations in the individual instances. For example, for the majority of people, higher value of both NPI and work-life conflict were correlated with higher perceived exposure risk. However, this increment was not linear and it varied from instance to instance. The NPI-compliance means more exposure for some compared to others, and we can observe the reverted relationship in some instances. Although it appears that people who felt more exposed tended to adopt more interventions, people who were not able to adopt the interventions might have felt more exposed to the disease.

Similarly, the general trend reveals that those with a higher level of work-life conflict to a larger extent evaluated their situations as being exposed, which may be due to being requested to meet physically for work, resulting in higher transmission risks. However, those who worked from home may have also faced increased demand of balancing work and family; however, this conflict did not necessarily reflect on the evaluation of their exposure risk as they were not required to go outdoors.

The same applies for gender and age. Although most women and younger people felt more exposed, there are observations that demonstrated the opposite prediction. In some instances, being a man and being older than 55 years indicated higher exposure risks. The variations were even larger when considering the predicted exposure risks based on the willingness to stay at home and living in Oslo in 2021. Those who felt more exposed tended to be more willing to stay at home. At the same time, those who preferred to go outside more often might have felt more threatened when the risk of contracting the disease outdoors increased. Although some Oslo residents felt more exposure risk, others felt safer. One explanation might be the large economic disparities and ethnic segregation within the city. While poorer people, students and minorities with fewer resources felt more exposed, those with higher social position were better able to avoid social distancing and, thus, reported lower exposure risk.

### Feature impact on model prediction

**Fig. 4:**
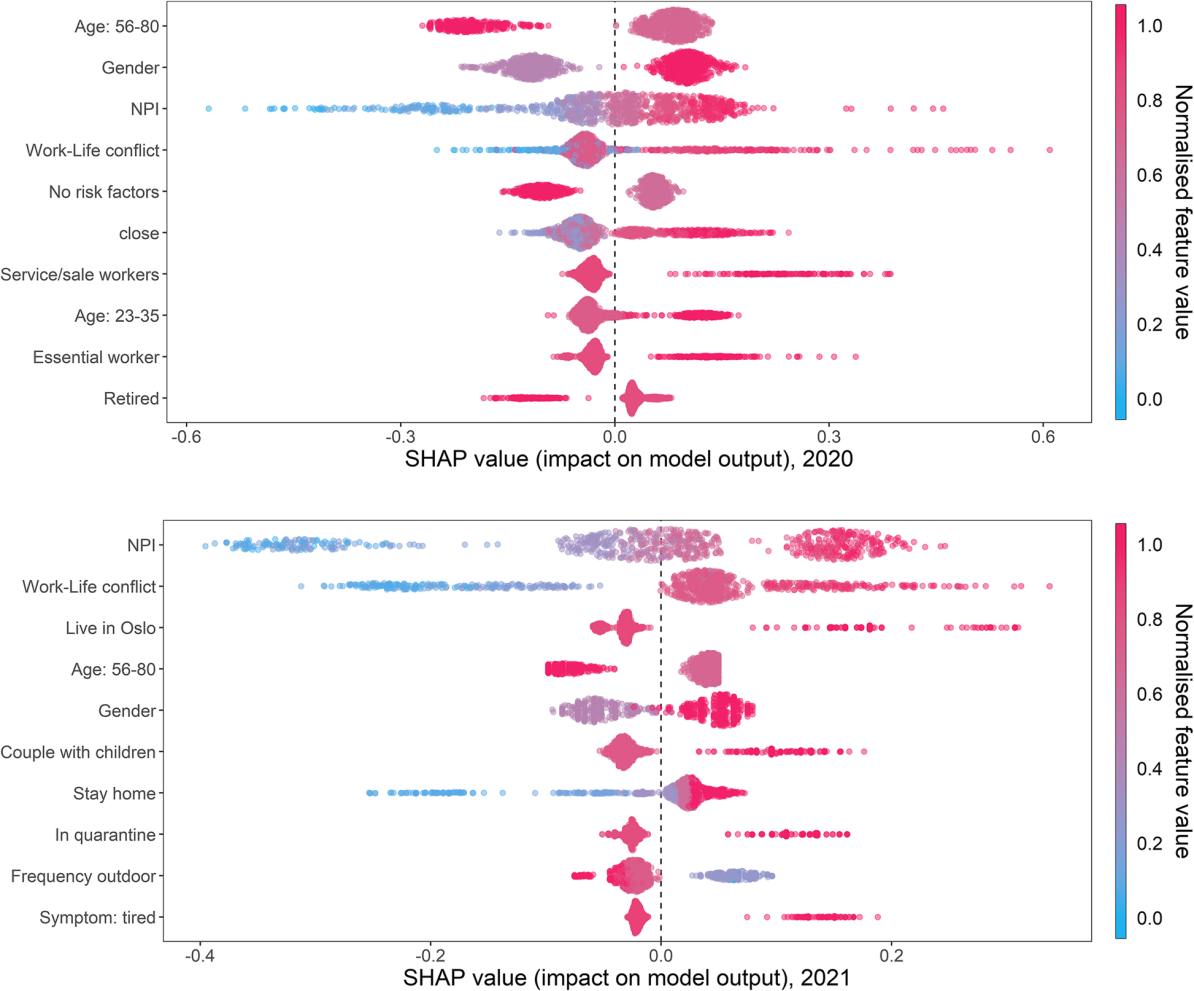
SHAP summary plot, 2020 and 2021

The next step was to explore the extent to which each individual feature contributed to predicting the total outcome of self-perceived exposure risk. Figure 4 displays the variable importance, feature effects and effect direction for each of the ten top-ranked impact features in 2020 and 2021 using the SHAP summary plot (see Additional file 1: Appendix E for plots with all the features).

In 2020, older age group (56–80 years) predicts lower perceived exposure, while younger age means more perceived exposure. Based on the absolute SHAP values (x-axis), the older observations have a higher impact on the model output than older adults. Being female indicates a higher prediction of perceived exposure risk, and the impact of being male on the model output is slightly higher than the former. The model prediction for exposure risk increases with a higher level of NPI adoption, however, the impact of the NPI feature is spread across a relatively wide range along the x-axis. The SHAP values also show that the higher values of work-life conflict have a larger impact on the prediction compared to instances of lower conflict.

In 2021, the direction and impact of the features are similar to that in 2020. In addition, living in Oslo increases the predictive value of perceived exposure to a large extent, and living in Oslo has a larger impact on the model output compared to living elsewhere. Willingness to stay at home and living in a household as a couple with children are also correlated with higher perceived risks, with the two variables showing a relatively large spread across the spectrum. Individuals who were not willing to stay at home impacted the predicted exposure risk more than people who were more willing to stay at home. The impact of households with couple and children on predicting perceived risks was greater than other types of households.

**Fig. 5:**
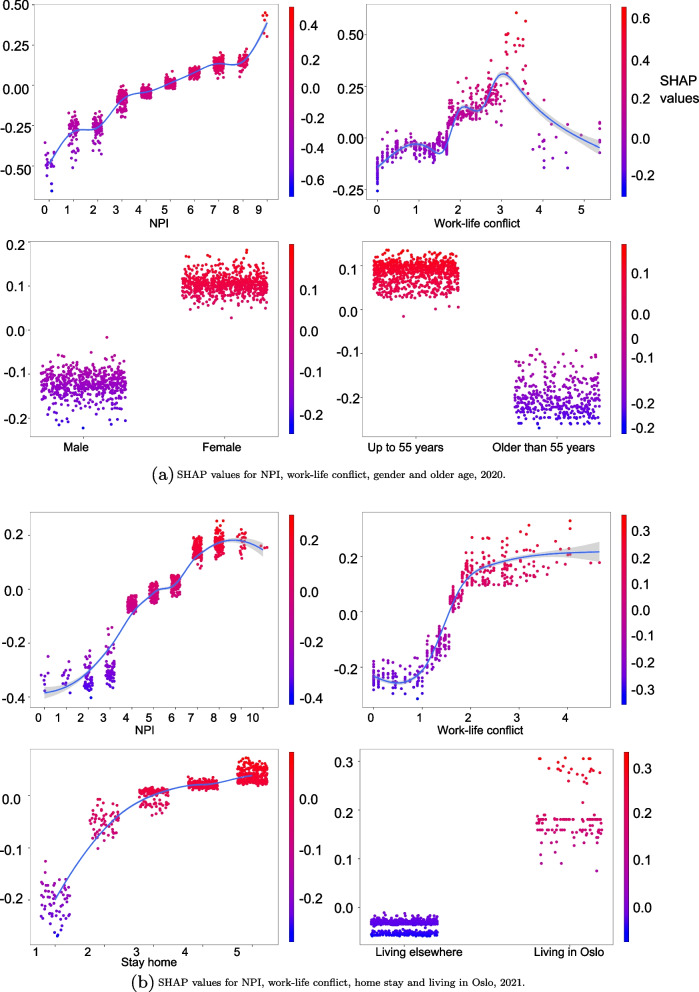
SHAP partial dependency plot predicting self-perceived exposure risk. **a** SHAP values for NPI, work-life conflict, gender and older age, 2020. **b** SHAP values for NPI, work-life conflict, home stay and living in Oslo, 2021

Zooming into the most interesting features for detailed information, Fig. 5 shows SHAP partial dependence plots for the four selected important variables in 2020 and 2021. In both the years, the importance of NPI-compliance varies mostly among individuals who adopted the fewest NPI-practices, followed by those who had the highest level of NPI follow-ups. For individuals in the middle, the importance of NPI on exposure prediction was rather homogenously low.

Heterogeneity in predicting perceived exposure risks among people with the highest levels of work-life conflict differed between the two periods. In 2020, the importance of work-life conflict increased exposure-prediction homogeneously to a certain point, after which the feature impact began to drop. For those who experienced the greatest imbalance between their work and life, work-life conflict no longer played an essential role in predicting perceived exposure. In 2021, the primary trend was increase in the feature importance due to a higher level of work-life conflict; we also observe greater differences in the feature impact for both individuals with the highest and the lowest levels of work-life conflict.

In 2020, the exposure prediction is particularly variable for older adults, but rather homogeneous for younger people. The age variable did not play an important role in predicting perceived exposure for those who were younger than 56 years. For those who were older than 56 years, the age variable was an important exposure-prediction for some, but not for all. When considering the gender variable, the degree of heterogeneity for variable impact was higher among men than women. In 2021, the role of residence area on exposure prediction varied greatly among people living in Oslo, but homogenously less important for people living elsewhere. Finally, the impact of willingness to stay at home on the exposure predictions had greater variations among those who were less willing to do so. Among those more willing to stay at home, this variable homogenously became less important.

### Additional checks

When running four different machine learning models, the models yielded robust results. The results indicate similar predictive performances and reveal more or less the same top ranked features. We also performed several additional analyses. Additional file 1: Appendix D.1 presents the partial dependence plots (PDP) illustrating the relationship between self-perceived exposure risk and the 10 most important features for each year. Additional file 1: Appendix D.2 plots the overall relationship between exposure and some of the most important variables, in both of the years, using partial dependence plots (PDPs). The results correspond with the findings from both ICE curves and SHAP plots. Generally, a higher level of NPI, work-life conflict, being female, being older (56–80 years), living in Oslo and willingness to stay at home were correlated with a higher predicted exposure risk.

Moreover, we present six randomly selected instances in each year, their top 10 explanatory features and the contribution of each feature to the predictions made locally (Additional file 1: Appendix F). The findings corresponded to the patterns demonstrated in the main analyses. We also assessed the interactions between the key features (Additional file 1: Appendix G). Notably, in 2020, the impact of lower compliance on lower perceived exposure was stronger for other occupation groups compared to service and sale workers and essential workers. Lower NPI compliance was also less likely to result in high exposure, especially for people in the 23-35 age group. In 2021, the association between high NPI and exposure risk was greater for females and for couples with children. Regarding work-life conflict, in 2020, this feature was associated with a higher risk of exposure, particularly for younger people. In 2021, this feature more heavily impacted the perceived exposure for men with higher work-life conflict levels and for those who were less willing to stay at home.

## Discussion

In this study, we used the machine learning method to predict self-perceived exposure risks during the COVID-19 pandemic. Using GBM, ENR, SVM and KNN algorithms, we found that the GBM model exhibited the best predictive performances for both 2020 and 2021. Based on the GBM, we identified the most important contributors to the prediction of perceived exposure risk. The general trend, for both years, noted that NPI and work-life conflict played the most essential roles in the degree of self-perceived exposure. Increased exposure was associated with a higher levels of recommendation compliance and work-life conflict. Younger age and female gender were also important features in predicting perceived exposure, especially in 2020. There were variations between the two years when considering other important predictors as well. In 2020, work and occupation emerged as the most important features. Working in the services and sales sectors and being an essential worker were strongly correlated with a high level of self-perceived exposure risk. In 2021, geographic location, household composition, epidemiological and intentional factors were found to be more important in predicting higher perceived risks.

As the pandemic entered different phases, institutions and individuals have had to adjust their ways of organising life and work. Perceptions on exposure risk are not entirely predetermined by cognitive abilities or personalities, but are rather a combination of sociodemographic background, life situations, possibilities of compliance and decisions taken when interpreting the COVID-19 situation. In 2020, remote work had not become normal praxis yet and routines of disease prevention in work environments were not sufficiently established. Therefore, self-perceived exposure was greatly determined by work and occupation almost uniformly. After a year of adjustments and lockdowns, new significant predictors emerged in 2021, most of them related to living situations—where people live, who they live with and their willingness to stay at home or going out. People’s actions are continuously producing and configuring structural conditions. Self-perceived exposure too was reproduced before the pandemic, transformed during the crisis and will potentially change into new forms after the crisis.

### NPI follow-ups

We found that while the middle levels of NPI follow-ups were homogeneously less important in predicting exposure, the importance of NPI in predicting exposure varied more among individuals with the lowest and highest degrees of NPI adaptation. People may have different needs, their behavioural patterns vary over the life course and across arenas, and there are different reasons why people comply [24]. For some, the level of compliance is correlated with fear of the coronavirus [15]. For others, however, compliance reflects their life situation—whether they are able to follow recommendations [1]. Therefore, the variables which played more important roles in predicting perceived exposure differed across individuals.

Varied dynamics and interactions might exist within different fields, and mitigation measures may present varying scopes and possibilities for different individuals during different phases of the pandemic. In 2020, the impact of lower NPI compliance on lower perceived exposure was observed to be stronger for other occupation groups than service and sale workers and essential workers. For these people, lower compliance was also more likely to correlate with higher levels of self-perceived exposure, compared to other occupations. Since these people were more likely to contract the disease through work and face-to-face interactions, following nonpharmaceutical recommendations more carefully would be perceived as a necessary intervention to keep themselves safe. In 2021, the dynamic altered, kindergartens opened up in Norway to relieve some burden from parents who work remotely. Following the contextual changes, the association between exposure and NPI became especially high among couples with children. Since children could potentially carry the virus when they return from kindergarten, it could have led parents to follow pandemic recommendations more carefully as important interventions to reduce exposure risks.

### Work-life conflict

Furthermore, the balance between work and daily life has been central to predicting self-perceived exposure risk in both the earlier and later phases of the pandemic. A general trend was that a higher level of work-life conflict was associated with more perceived exposure risk. Higher work-life conflict implies more demands at work; for example, physical presence requested at work or being constantly available when working remotely [62]. Greater work-life conflict has also been associated with more psychological distress [25]. On the one hand, work-life conflict may imply meeting up in person at work for workers in services and sales and the younger age group. Significant conflict between work and life might, therefore, indicate pressure from being exposed to more disease-contracting situations, less abilities for social distancing and, hence, greater perceived exposure risks. On the other hand, high work pressure for remote workers may keep people more at home to work and, thus, minimise their social contact. Therefore, for remote-workers with high level of work-life conflict, some other factors might also play a greater role in predicting perceived exposure to COVID-19. This might explain why higher level of work-life conflict became less important particularly for women, as they tend to take a larger share in childcare and household work then men.

### Demographics

Self-perceived exposure risks are based on perceptions of one’s vulnerability and evaluations of current life situations. Such perceptions are at the same time affected by expert definitions of ‘at risk’ groups and one’s previous experiences [22]. Older adults reported less exposure risks compared to younger people. Older age correlates with higher clinical probability of becoming severely ill, and such information from health authorities may influence their choice and behaviour of being more careful in daily life. At the same time, older adults also have more opportunities for social distancing, thus limiting disease contraction.

It is worth noting the gender dimension when considering exposure and age. While low exposure risk was particularly impactful for older female adults, the reverse effect—higher exposure—coupled with young age was also especially clear in the case of younger women. At the same time, the impact of age on perceived exposure was rather homogenous among younger people, meaning that the impact of age was uniformly stronger for younger women than younger men on exposure risk perception. This is consistent with previous studies. Scholars located gender differences in perceived risks, as they are construed differently for women and men, both socially and culturally [63]. Younger female adults, burdened with work and care-provider obligations, carried a greater concern and mental burden during the COVID-19 pandemic compared to younger men.

### Study limitations

This study has several limitations. First, although the presented variables are identified as the most important predictors, there might be other interesting relationships to uncover in future studies. For example, previous experience of similar crisis might play an essential role in understanding how people evaluate their own situation and perceive the ongoing pandemic. Psychometric instruments and ethnic origins are also useful variables for predicting exposure outcomes. Unfortunately, the CorLife datasets did not have such information. Future studies that include such variables may achieve higher model performance. Second, due to the aim and scope of the paper, we did not examine the causal relationships between self-perceived exposure risks and its predictors. Many of them might have a reversed causal relationship with perceived exposure. Future studies into such relationships can contribute to in-depth understanding of risk perceptions. It would also be interesting to examine perceived risks related to different social arenas. Further development of a more comprehensive indicator of exposure risk could also merit future research.

### Study implications, generalisability and policy implications

Aside from understanding the disease agent itself, understanding people’s perceptions of exposure risk and their responses to the pandemic is crucial for comprehending and navigating the COVID-19 pandemic [64, 65]. Perceptions of exposure risk not only correlate with preventive behaviours and public health compliance [66] but are also amongst the most important predictors of mental illness and life satisfaction [6, 8, 9]. Individuals who perceive themselves to be more at risk and those with higher health anxiety are more likely to report fear [41] and to adhere to protective measures in epidemics [42]. The self-perceived exposure risk and the fear of being exposed to the disease can also influence key attitudes and individual virus-mitigating behaviours during a pandemic [15, 28]. Therefore, there is an urgent need to systematically predict self-perceived exposure risks, to identify the social groups at risk and to detect the most important predictors of such risks.

This study has several implications. This paper contributes to the relatively small amount of predictive research in social sciences with a focus on predicting self-perceived exposure; it also adopts a broader definition of exposure by including a broad range of social, behavioural and epidemiological factors in predicting important outcomes. Risk perception is intertwined with people’s daily activities, contextual situations and socioeconomic resources. Identifying vulnerable social groups and important contributors to a higher risk of exposure in relation to complex social realities is essential to develop future mitigation policies. In a similar study, scholars developed decision-making frameworks to analyse COVID-19-related transmission vulnerability in different cities based on clusters of numerous factors related to the climate, hygiene/safety, decision-making responsiveness and sociodemographic, economic and psychological variables [67]. Similarly, by incorporating multiple features into the analysis while acknowledging individual heterogeneities, the findings in this study can provide valuable insights and assist in decision-making processes. The results may be useful for adjusting interventions that target various social groups in different pandemic phases in Norway, which could directly benefit the most vulnerable people.

Such interventions may include, for example, workplace policies that address work-life balance and the implementation of flexible working arrangements to minimise the need for employees to be physically present in crowded workplaces; public and community engagement to promote social support and provide resources such as mental health support, assistance with accessing essential goods and services amongst more vulnerable populations; and the promotion of home-based activities which provide support and resources to individuals to facilitate productive and enjoyable activities at home.

However, policy recommendations should also consider evidence amongst other sources, including expert opinions, scientific studies and public health guidelines. Moreover, machine learning models trained on data from Norway have the potential to be generalised to some extent, but it is important to consider the context and limitations of their applicability. Since the training data used to develop the models primarily represents demographic and social conditions within Norway, the models’ generalisability may be limited to similar populations. The effectiveness of machine learning models can be influenced by social context. Predictors of the self-perceived risk of exposure to COVID-19 identified within the Norwegian data primarily represent demographic and social conditions within Norway. Therefore, caution should be exercised when applying policy recommendations derived solely from the Norwegian context; they might not be generalisable to the same extent to other countries with different cultural, socioeconomic or healthcare systems.

Nevertheless, while the direct transfer of a model trained on Norwegian data to another country may not be ideal, transfer learning techniques can be employed. Transfer learning allows models to leverage knowledge gained from one context to improve performance in another. By retraining the model with additional data from the target country or region, its generalisability can be enhanced. Furthermore, to assess the generalisability of the machine learning models, it is crucial to validate the model’s performance using external datasets from different regions or countries. This process can help determine whether the identified predictors remain consistent across diverse populations or whether additional variables need to be considered.

## Conclusion

One important way to enhance pandemic preparedness is by increasing the understanding of people’s self-perceived exposure in response to such events. There is, therefore, a need to conduct studies on perceived exposure risk, explain why some factors predict such risks more strongly and interpret how predictions vary among individuals and social groups.

The prediction of exposure perception depends on the level of compliance to interventions and work-life balance to a large extent. The relationships are non-linear and vary among people with different sociodemographic backgrounds in diverse work and living conditions and different phases of the pandemic. Therefore, future policies and interventions should be adapted to the needs of people from various life situations.

Since the COVID-19 pandemic posed different challenges to people of different socioeconomic statuses, it is essential to gain information about the most vulnerable social groups to develop relevant social policies and interventions. The predictive model in our study may help policymakers and stakeholders forecast vulnerability and risk factors. By integrating analyses that both identify social determinants and predict outcomes, the machine learning models in this study can contribute to the early detection of vulnerable groups and help implement timely interventions targeting people from different social groups.

## Supplementary Information

**Additional file 1.** Appendices.

## Data Availability

The data and materials are only available for persons involved in this project (No. 126 127). Requirement of data and materials access must to be sent to REK for approval. Contact person: Nan Zou Bakkeli, e-mail: nan.bakkeli@oslomet.no.

## Abbreviations

COVID-19: Coronavirus disease of 2019
ENR: Elastic net regularisation
GBM: Gradient boosting machine
ICE: Individual conditional expectation
KNN: K-nearest neighbour
MAE: Mean absolute error
NPI: Nonpharmaceutical intervention
PDP: Partial dependent plot
RMSE: Root mean square error
RSQ: Coefficient of determination
SHAP: Shapley additive value
SVM: Support vector machine
