# Supplementary material for: Predicting COVID-19 exposure risk perception using machine learning
Source: BMC Public Health. 2023 Jul 18;23:1377. doi: 10.1186/s12889-023-16236-z (PMC10353112; doi:10.1186/s12889-023-16236-z)
Supplement: Supplementary file 1 — Additional file 1. Appendices. [file 12889_2023_16236_MOESM1_ESM.pdf]

Appendices

Appendix A. Literature overview: relevant previous studies

Authors	Title	Journal	Method	Findings
Bakkeli (2022)	Predicting Psychological Distress During the COVID-19 Pandemic: Do Socioeconomic Factors Matter?	Social Science Computer Review	Machine learning models	Exposure risk as the most important predictor of mental illness and depression
Chen et al. (2021)	Excess mortality associated with the COVID-19 pandemic among Californians 18-65 years of age, by occupational sector and occupation: March through November 2020	PLOS ONE	Time-series analysis: dynamic harmonic regression models	Latinos in the food or agriculture sector, black people in the transportation or logistics sector, and white people in manufacturing facilities had the highest mortality rates
Harper et al. (2021)	Functional Fear Predicts Public Health Compliance in the COVID-19 Pandemic	International Journal of Mental Health and Addiction	Linear regression models	Protective behaviour during COVID-19 was not influenced by political orientation or moral perceptions, but was more often caused by fear
Jørgensen et al. (2021)	Fear of COVID-19, compliance with recommendations against virus transmission, and attitudes towards vaccination in Sweden	Heliyon	Logistic regressions	The Fear of COVID was significantly associated with compliance with all COVID-19 recommendations and with mental health. Fear of exposure is higher for women, those have had mental health treatment, perceived to be at risk and those who are not working or study
Lanciano et al. (2020)	Risk Perceptions and Psychological Effects During the Italian COVID-19 Emergency	Frontiers in Psychology	ANOVA	Cognitive and affective risk-related variables contributed to explain the several risk perception domains differently
Li et al. (2020)	Clinical and pathological investigation of patients with severe COVID-19	JCI Insight	Descriptive statistics and statistical tests	Identified various epidemiological and pathological determinants, and clinical and laboratory characteristics

Lu et al. (2020)	Psychological status of medical workforce during the COVID-19 pandemic: A cross-sectional study	Psychiatry Research	Chi-square test, rank-sum test and ordinal logistic regressions	The severity of fear, anxiety and depression were significantly different between medical and administrative staffs. Front-line medical staff with close contact with infected patients were likely to feel fear and to suffer anxiety and depression
Muller et al. (2020)	The mental health impact of the covid-19 pandemic on healthcare workers, and interventions to help them: A rapid systematic review	Psychiatry Research	Systematic review	Exposure to covid-19 was the most commonly reported correlate of mental health problems
Ni et al. (2020)	Mental Health, Risk Factors, and Social Media Use During the COVID-19 Epidemic and Cordon Sanitaire Among the Community and Health Professionals in Wuhan, China: Cross-Sectional Survey	JMIR Ment Health	Logistic regression analysis	Close contact with individuals with COVID-19 and spending ≥ 2 hours daily on COVID-19 news via social media were associated with probable anxiety and depression. Social support was associated with less probable anxiety and depression
Niño et al. (2021)	Race and ethnicity, gender, and age on perceived threats and fear of COVID-19: Evidence from two national data sources	SSM - Population Health	Logistic regressions	Race and ethnicity, gender, and age play a significant role in shaping threat and fear perceptions of COVID-19
Pijls et al. (2021)	Demographic risk factors for COVID-19 infection, severity, ICU admission and death: a meta-analysis of 59 studies	BMJ Open	Meta-analyses	Men and patients aged 70 and above have a higher risk for COVID-19 infection, severe disease, ICU admission and death

Prasad et al. (2021)	Prevalence and correlates of stress and burnout among U.S. healthcare workers during the COVID-19 pandemic: A national cross-sectional survey study	EClinical Medicine	Multilevel linear regression and ordered logistic regression	The prevalence of fear of exposure or transmission was higher among nursing assistants and black and Latino workers than white workers and higher among women than men
Proto and Quintana-Domeque (2021)	COVID-19 and mental health deterioration by ethnicity and gender in the UK	PLOS ONE	Short and long regressions	Both women and Black, Asian, and minority ethnic men experienced a higher average increase in mental distress than White British men
Thomas et al. (2022)	A descriptive analysis of 2020 California Occupational Safety and Health Administration covid-19-related complaints	SSM - Population Health	Descriptive statistics and chi-square tests	Exposure to COVID-19 disproportionately affects specific occupations and industries in which essential workers are more likely to be exposed
Wu et al. (2021)	Two tales of one city: Unequal vulnerability and resilience to COVID-19 by socioeconomic status in Wuhan, China	Research in Social Stratification and Mobility	Logistic regression	Higher socioeconomic status (SES) is associated with a lower risk of infection. Higher SES reduces mental distress during the pandemic, and this is particularly strong for those who contract the virus or who have family members infected with the disease

Appendix B. Data and feature variables

Data

The CorLife-surveys from 2020 and 2021 aimed to capture important socioecoonmic, behavioural and epidemiologic factors in Norway during the COVID-19 pandemic. The data included information on (1) COVID-19-related epidemiologica factors such a list of infection symptoms, potential test results for coronavirus and health complications and risk groups; (2) psychological distress and loneliness; (3) COVID-19 protective behaviour connected to nonpharmaceutical interventions; (4) work situation, occupation and work-life conflict; (5) social contacts, daily praxis and consumption; (7) technology use; and (8) socioeconomic, demographic and geographic background information.

The response rates for the three waves were 41%, 28% and 30%, respectively. The survey discrepancies were mainly caused by the panel maintenance process, which involved inviting new respondents and deleting non-responding individuals; most of the dropouts in the survey were random (Bakkeli, 2022). Based on previous analysis of Norwegian surveys using the same sampling method as the Corlife survey, Hellevik (2016) concluded that the sampling methods were robust, the nonrespondents had a random pattern and the results from such data should not be biased.

Feature variables

The study included 54 features to predict self-perceived exposure risk. They are socioeconomic and demographic background, work and occupation, behavioural features and COVID-19-related epidemiological factors.

Socioeconomic and demographic features included *age* (18–22, 23–35, 36–55, 56–80), *gender* (female=1), *geographic residence area* (with a population <2,000, 2,000–4,999, 5,000–50,000, >50,000 (but do not live in Oslo), and Oslo), *higher education* (college/university=1), *household income in Norwegian kroners (NOK)* (<300,000, 300,001–500,000, 500,001–800,000, 800,001–1,000,000, 1,000,001–1,500,000, >1,500,000) and *household types* (couple with children, couple without children, single parent, living alone and other types).

Work and occupational features included whether a person was *currently employed*, and, if so, which occupation the person had. Respondents' *occupations* were coded into ten major categories using the Norwegian Standard Classification of Occupations (STYRK-08), which was adapted from the International Standard Classification of Occupations 2008 (ISCO-08) (SSB, 2011). Respondents were placed into 10 major categories: managers, leaders and political leaders; professionals and academic professionals; technicians and associate professionals; clerical support workers; service and sales workers; skilled agricultural, forestry and fishery workers; craft and related trades workers; plant and machine operators, transport workers, assemblers; elementary occupations, such as cleaners and helpers; and others. Retirees, job seekers and those on sickness leave or receiving disability benefits were separately categorised. Also, people working within the healthcare sector, grocery stores, transportation, police and other essential services were identified as being *essential workers*. All the above-mentioned work-related variables were dichotomised into dummy variables.

The indicator of *work-life conflict* was constructed by using four variables, each ranging from 'never' to 'always': 'Work demands often interfere with family life'; 'Family responsibilities often interfere with my job'; 'I limit the time I spend working digitally when the working day is over'; and 'My employer shows little understanding for my

family and care responsibilities'. Principal component analysis was applied to generate the indicator, and a higher value indicated a higher level of work-life conflict.

To examine **behavioural features**, we constructed an indicator for respondents' adaptation of *nonpharmaceutical interventions (NPI)* (ranged from 0–10, in which 0 indicated the lowest degree of following nonpharmaceutical interventions, and 10 indicated the highest level of following all interventions. The variable was constructed based on whether the respondents had followed instructions, such as washing hands/using disinfectant constantly; using protective equipment, such as facemasks; generally being more careful with washing and cleaning; cancelling or postponing physical meetings or gatherings; cancelling or postponing travel; avoiding public gatherings and crowds; using fewer public transports than usual; keeping children at a safe distance from each other; and other interventions.

In addition, *mobility pattern* showed number of times respondents went outdoors during the past five days (never went out, once a day, 2–3 times a day, 4–5 times a day, more than 5 times a day). *Social distancing* was measured by asking respondents the number of times they had gone outdoors in the past five daysthe number of people they had been less than a meter away from during the past five days, except people from the same household (none, 1–3 persons, 4–6 persons, 7–10 persons, more than 10 persons). *Social contact* was measured by mapping how often the respondents had digitally contacted friends and relatives during the pandemic and the frequencies with which they were in touch with colleagues and others through work (several times an hour, several times a day, couple of times a day, once a week, less than once a week). There was also a measure of intention based on people's *willingness to stay at home* (ranged from least to most willing to stay at home in five categories).

Epidemiological features were measured by *COVID-19 disease risk factors* (being old, having underlying diseases, smoking, pregnancy, testing COVID-19 positive, isolating because of disease, in quarantine due to either suspected possible transmission or travelling) and *COVID-19-related symptoms* (coughing, heavy breathing, fever, muscle pain, diarrhoea and tiredness).

Imputation

We imputed missing values for variables as described in (Bakkeli, 2022). Imputation was done depending on the type of missing data mechanism. Discrepancies for occupation were grouped into an additional category, and missing values for income were filled in by using multinomial logistic regression imputation methods. The observed cases' mean value was used as a substitution for missing values of social contact and work-life conflict. Discrepancies for education were filled by using hot deck imputation, where missing values were replaced by the most similar cases based on gender, occupation and income level.

Appendix C. Distribution of variables in 2020 and 2021

C.1. *Density distribution of continuous variables, 2020 and 2021*

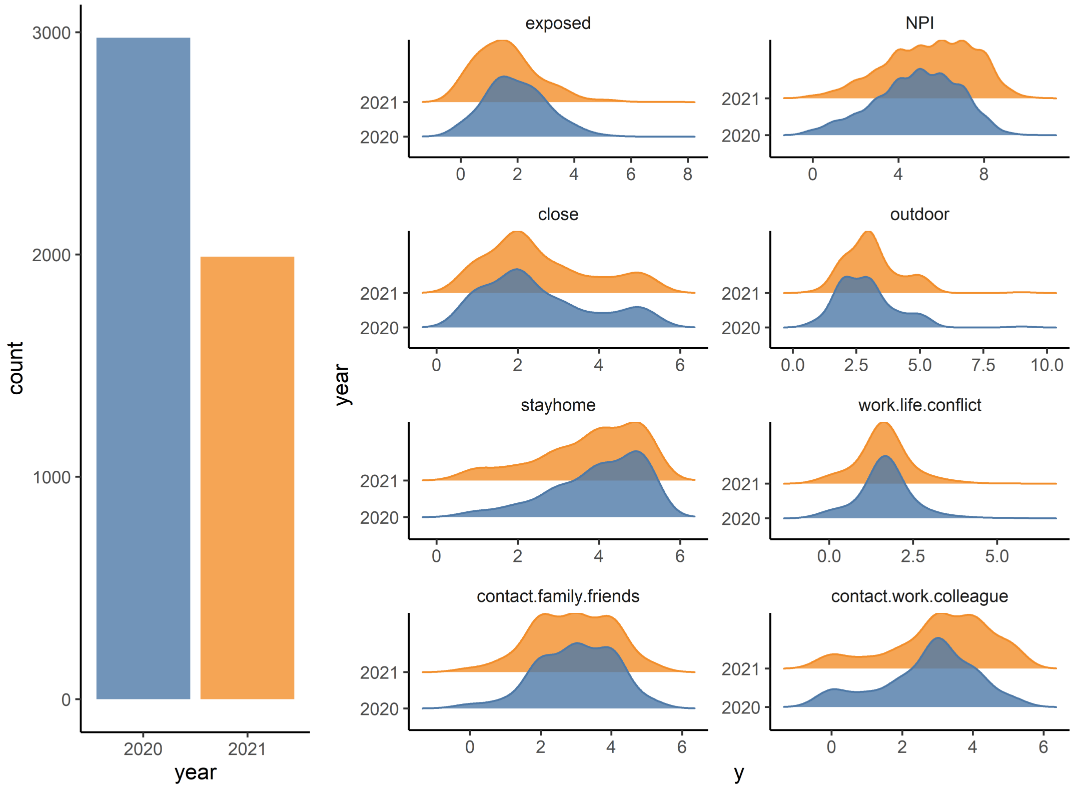

C.2. *Distribution of social demographic features, 2020 and 2021*

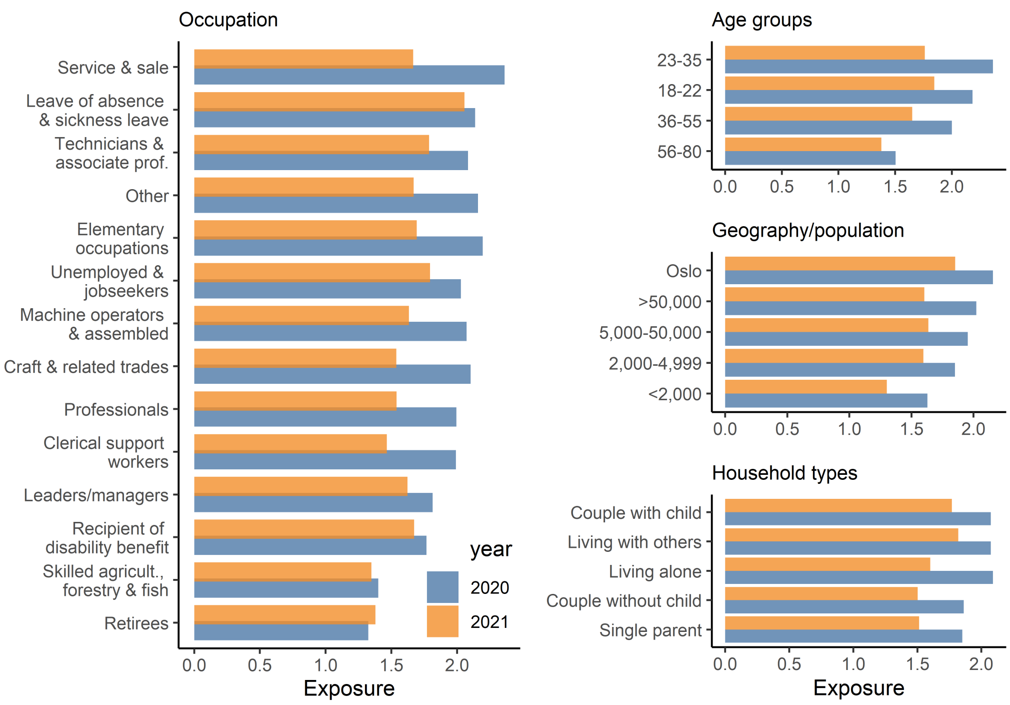

C.3. *Distribution of social epidemiological features, 2020 and 2021*

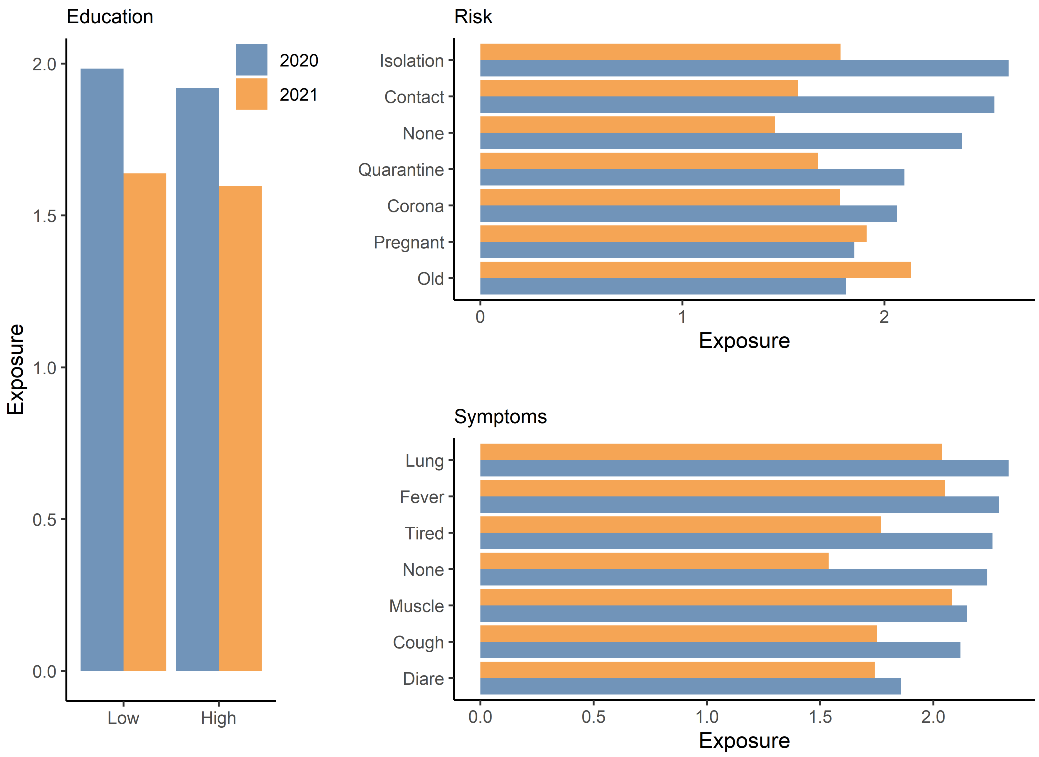

Appendix D. Partial dependence plots, 2020 and 2021.

D.1. *Partial dependence plots for the ten most important variables, 2020 and 2021.*

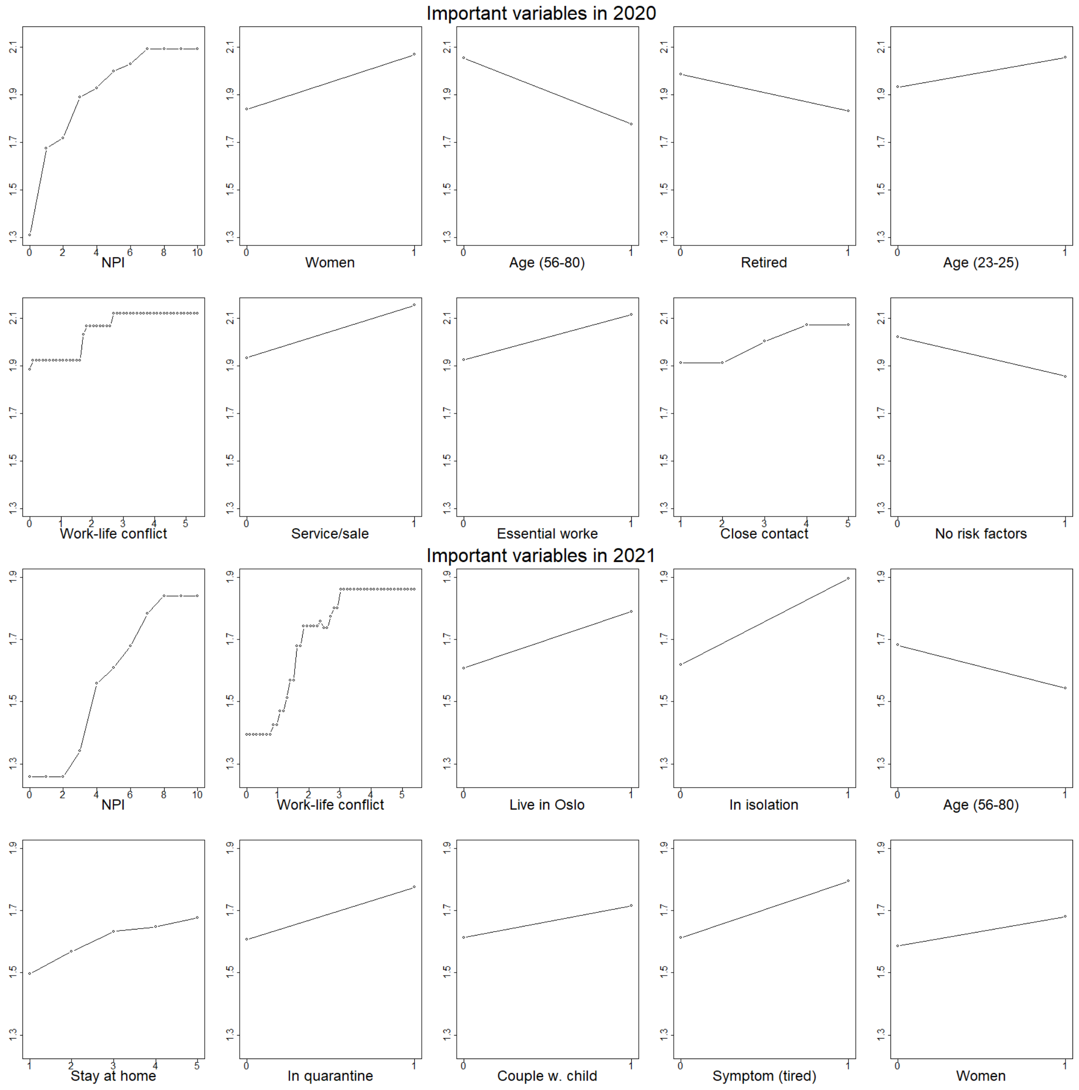

D.2. *Partial dependence plot for predicting self-perceived exposure risk, selected variables.*

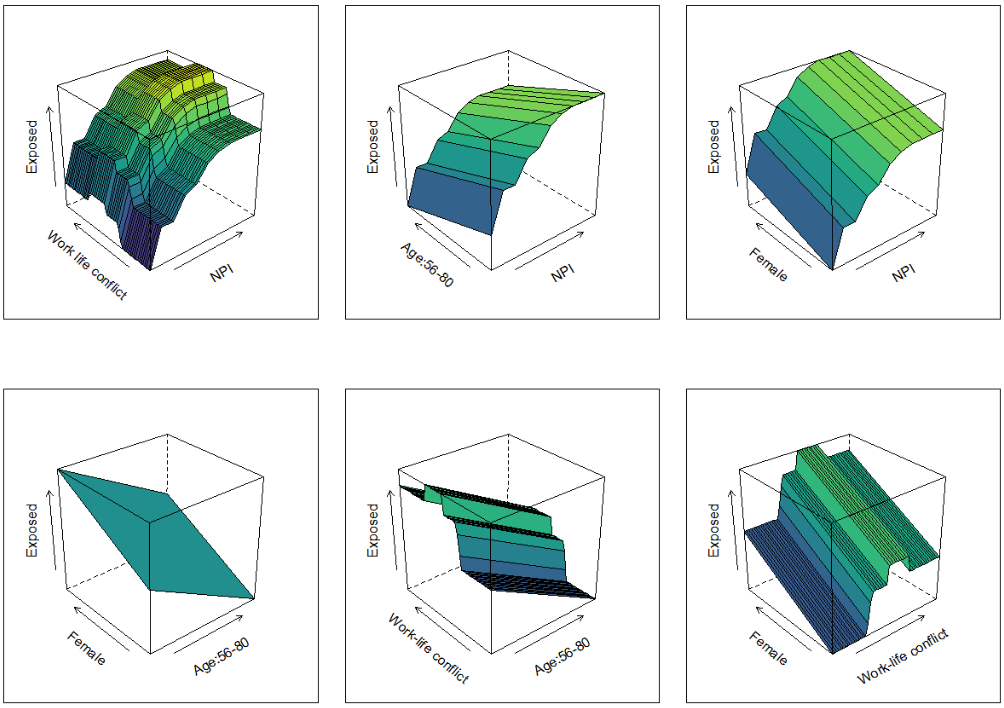

(a) Perceived exposure by NPI, work-life conflict, gender and older age, 2020.

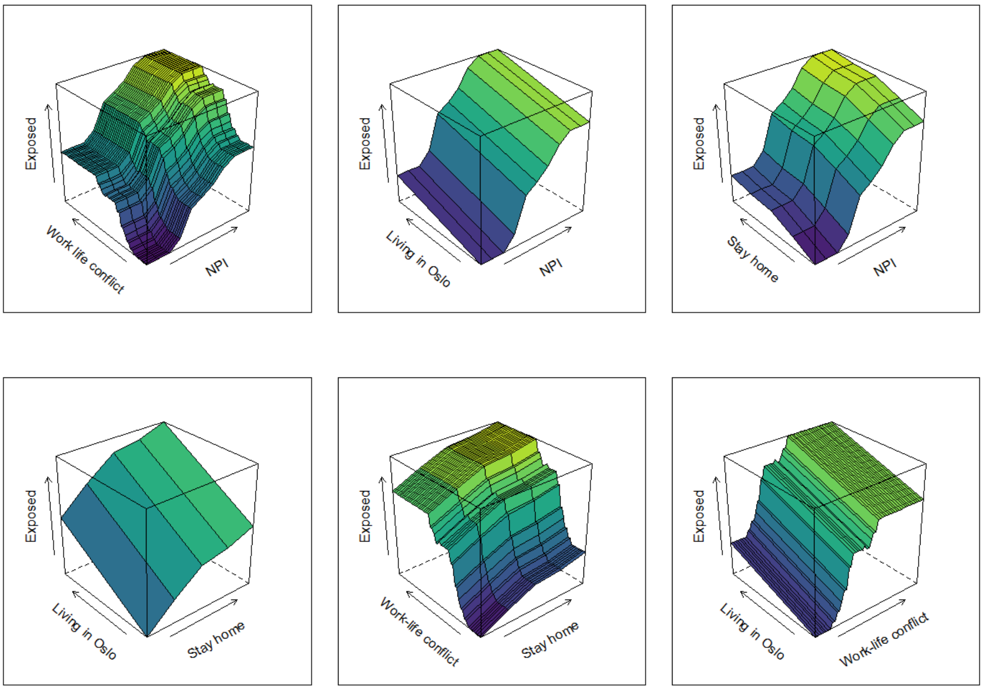

(b) Perceived exposure by NPI, work-life conflict, home stay and Oslo, 2021.

Appendix E. Shapley additive explanations (SHAP) summary plot, 2020 and 2021

E.1. *SHAP summary plot, 2020.*

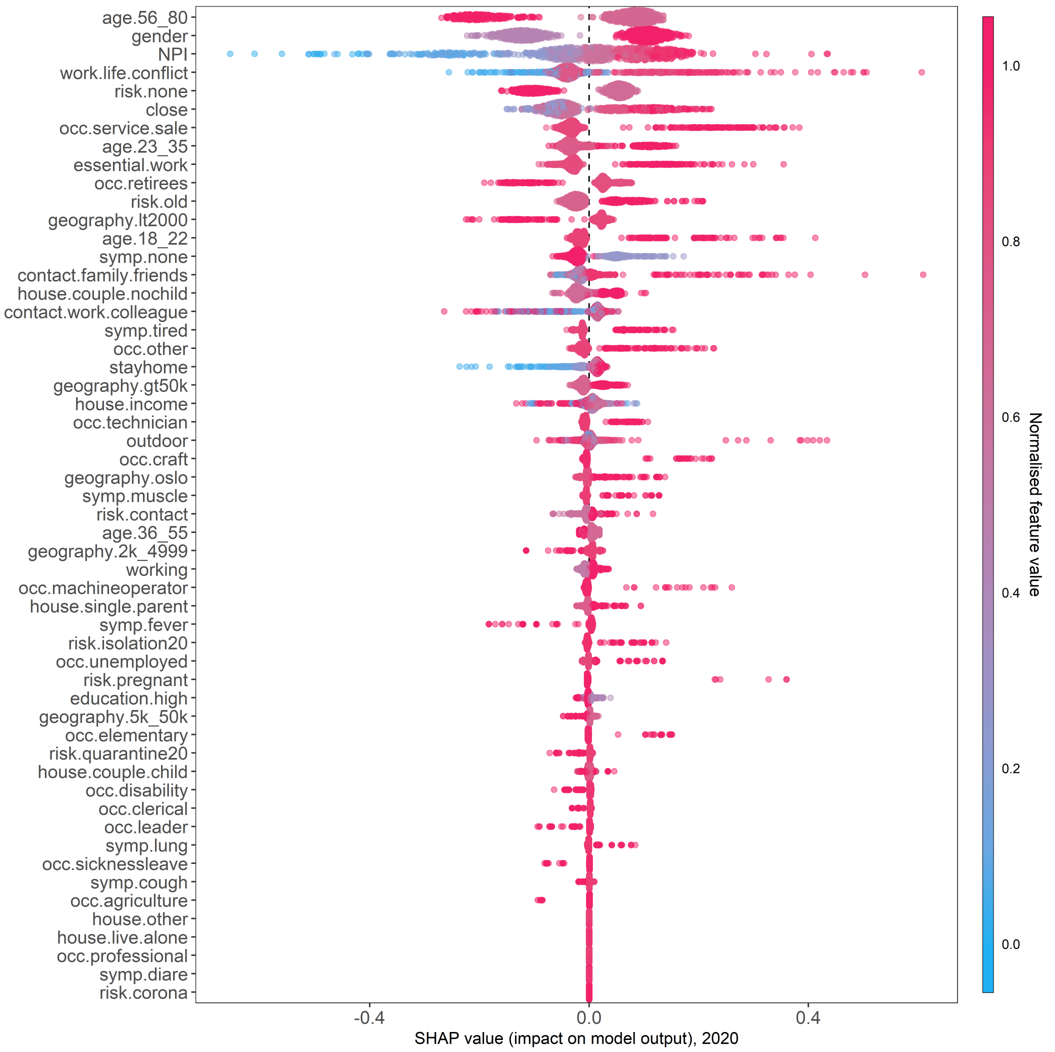

E.2. *SHAP summary plot, 2021.*

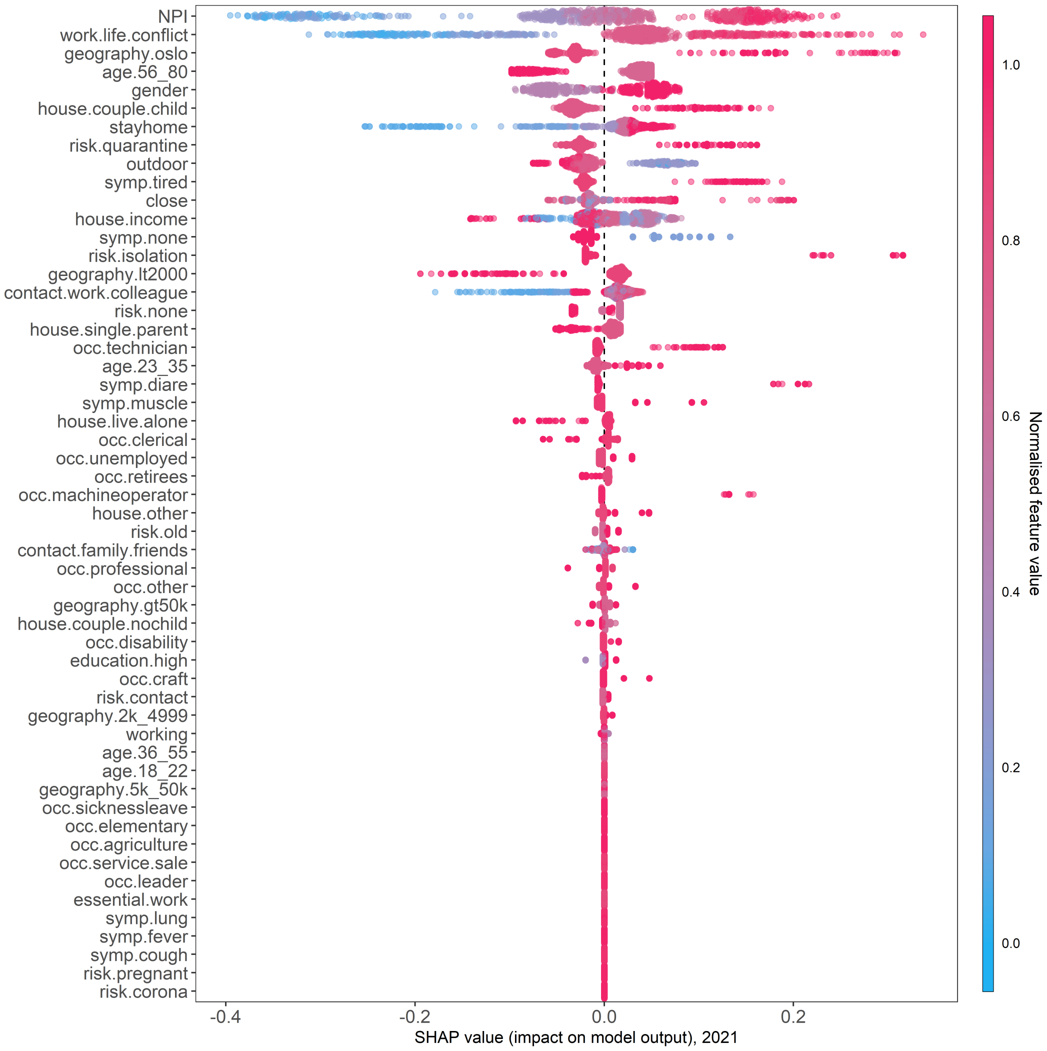

Appendix F. Local interpretable model-agnostic explanations

F.1. *Local interpretable model-agnostic explanations, 2020*

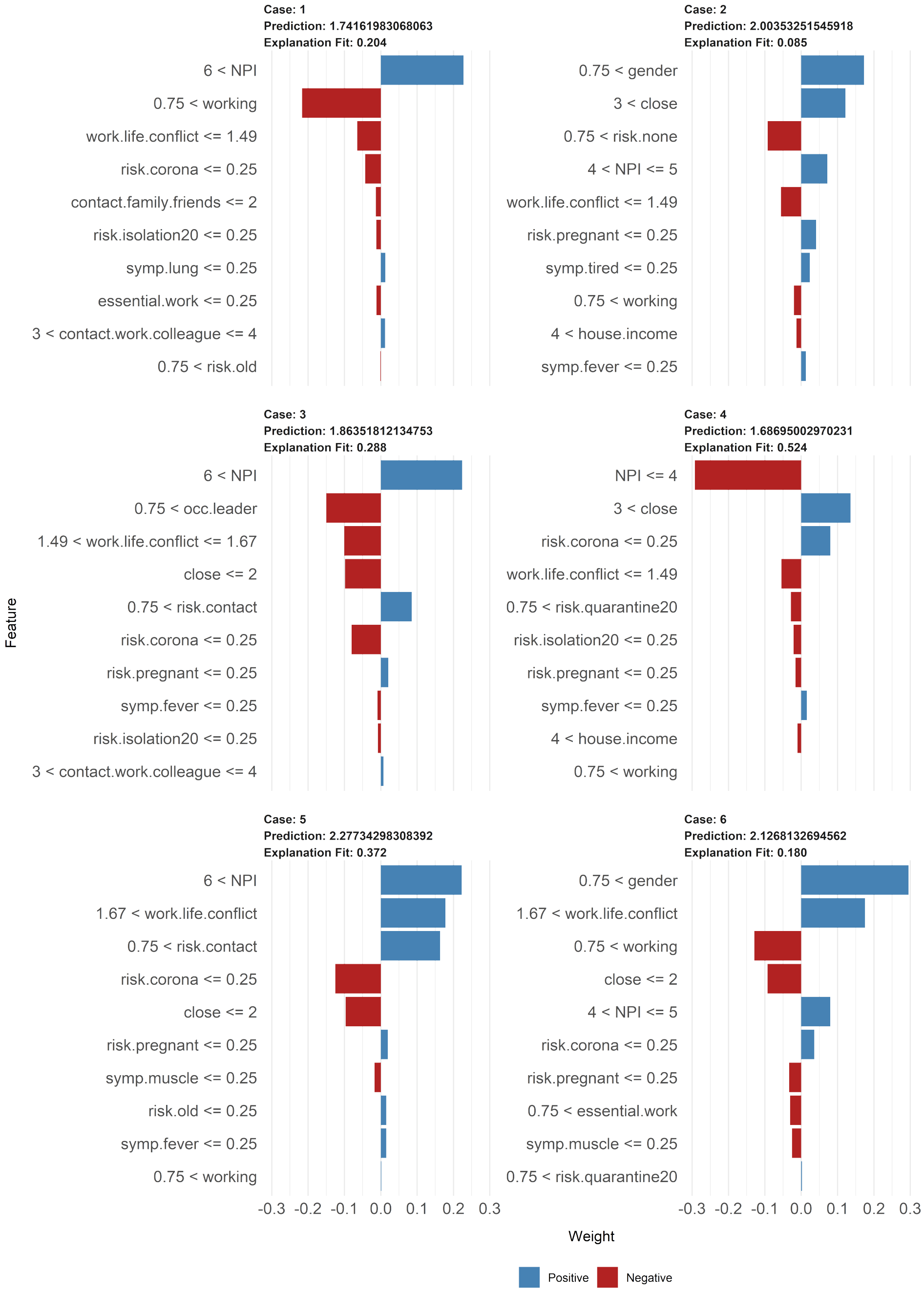

Top ten important variables in six random cases in 2020. Case four has the highest explanation fit (.524). The top three influencing variables are NPI (NPI<4 decreases exposure risks), number of people in close distance (>3 people increases exposure risks), and tested COVID-19 positive (not infected) increases exposure risk.

F.2. *Heat map for local interpretable model-agnostic explanations, 2020*

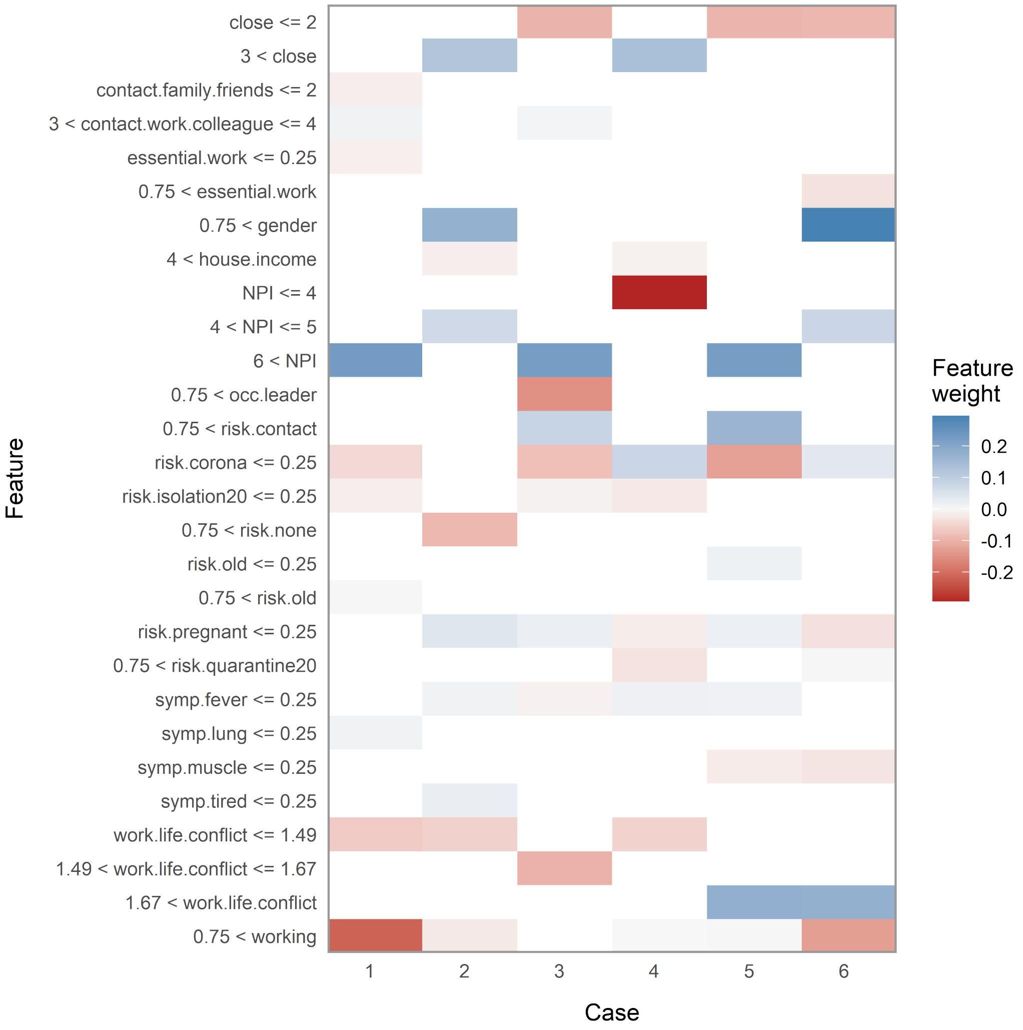

Top ten important variables in six random cases in 2020. The heat map shows how the different features influence each case. The common feature that positively influenced (higher weight) the exposure risk across three of six observations was having an NPI score higher than 6. Having tested positive for COVID-19 had a different prediction effect among the observations. For a transmission probability lower than .25 (‘risk.corona<=.25’), two observations showed increased prediction values of being more exposed (blue), but three felt less exposed (red).

F.3. *Local interpretable model-agnostic explanations, 2021.*

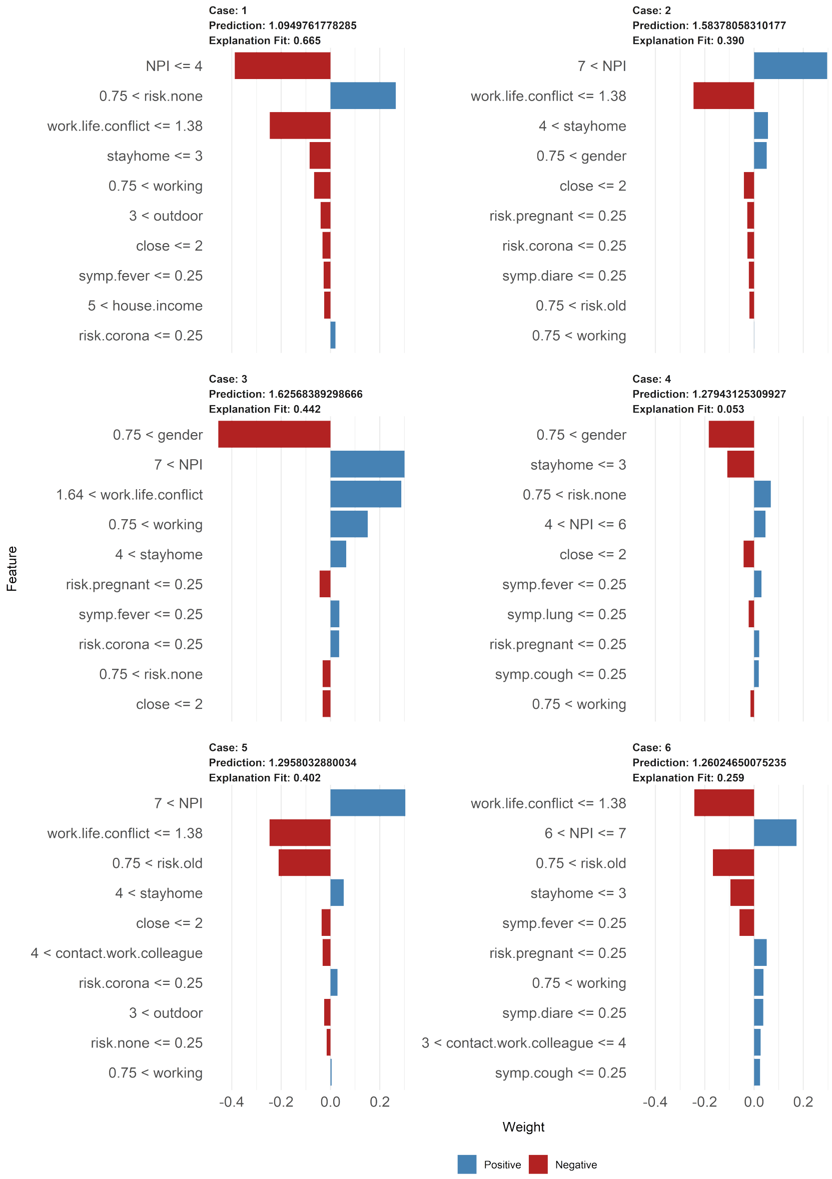

Top ten important variables in six random cases in 2021. Case one has the highest explanation fit (.665). The top three influencing variables are NPI (NPI<=4 decreases exposure risks), having no risk factors of contracting the disease (>.75) increases exposure risks and having lower work-life conflict decreases exposure risk).

F.4. ***Heat map for local interpretable model-agnostic explanations, 2021.***

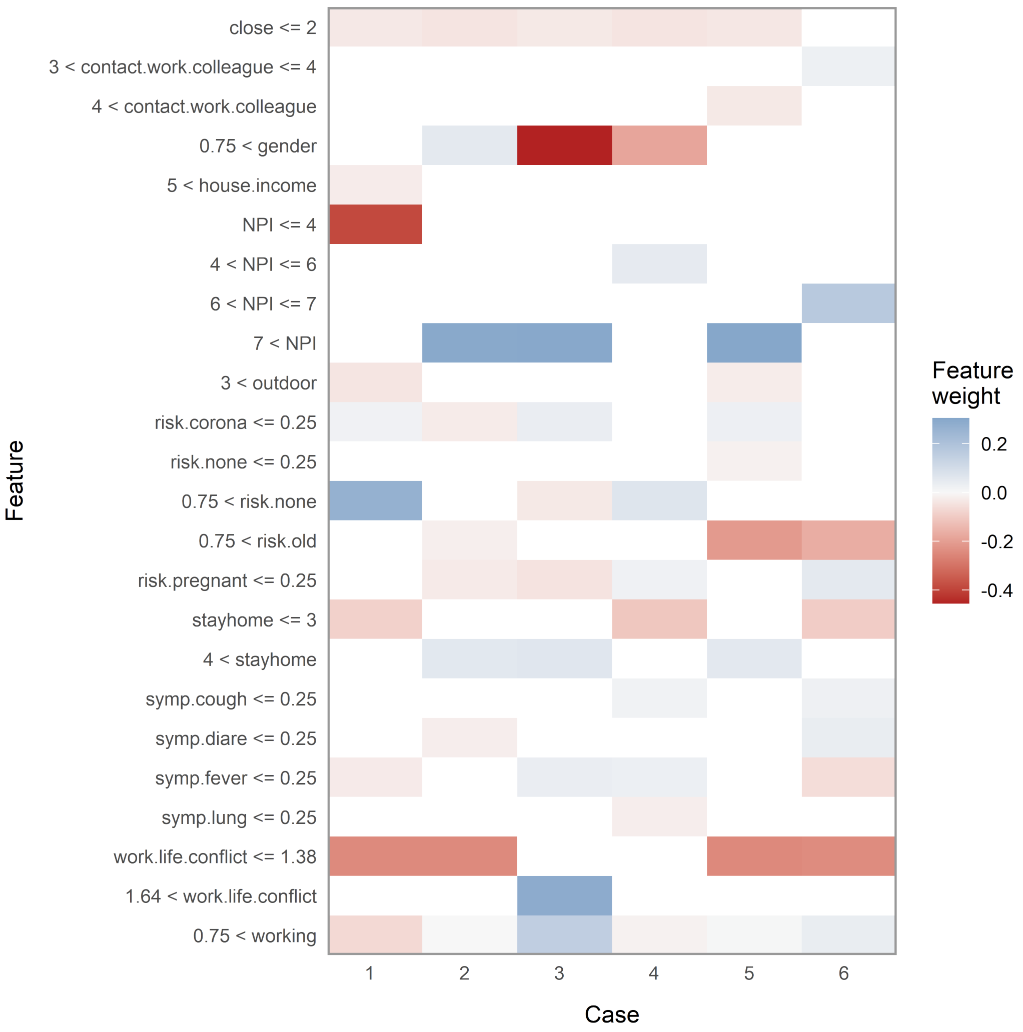

Top ten important variables in six random cases in 2021. The heat map shows how the different features influence each case. The common feature that positively influenced (higher weight) the exposure risk across three of six observations was having an NPI score higher than 6. Having lower work-life conflict (‘work.life.conflict<=1.38’) indicated lower exposure for four in six observations.

Appendix G. SHAP dependence plot

G.1. *Interaction between NPI and key features in 2020.*

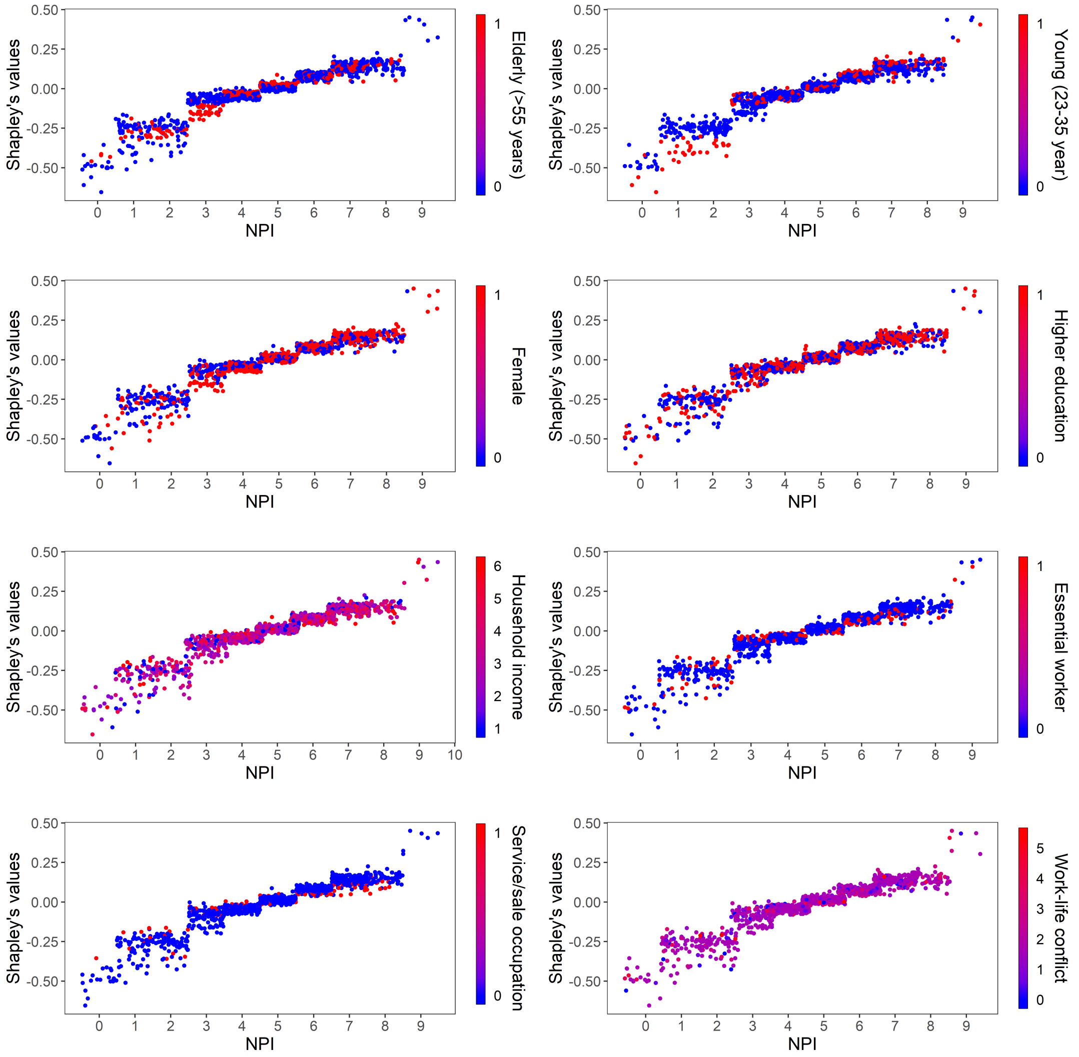

G.2. *Interaction between work-life conflict and key features in 2020.*

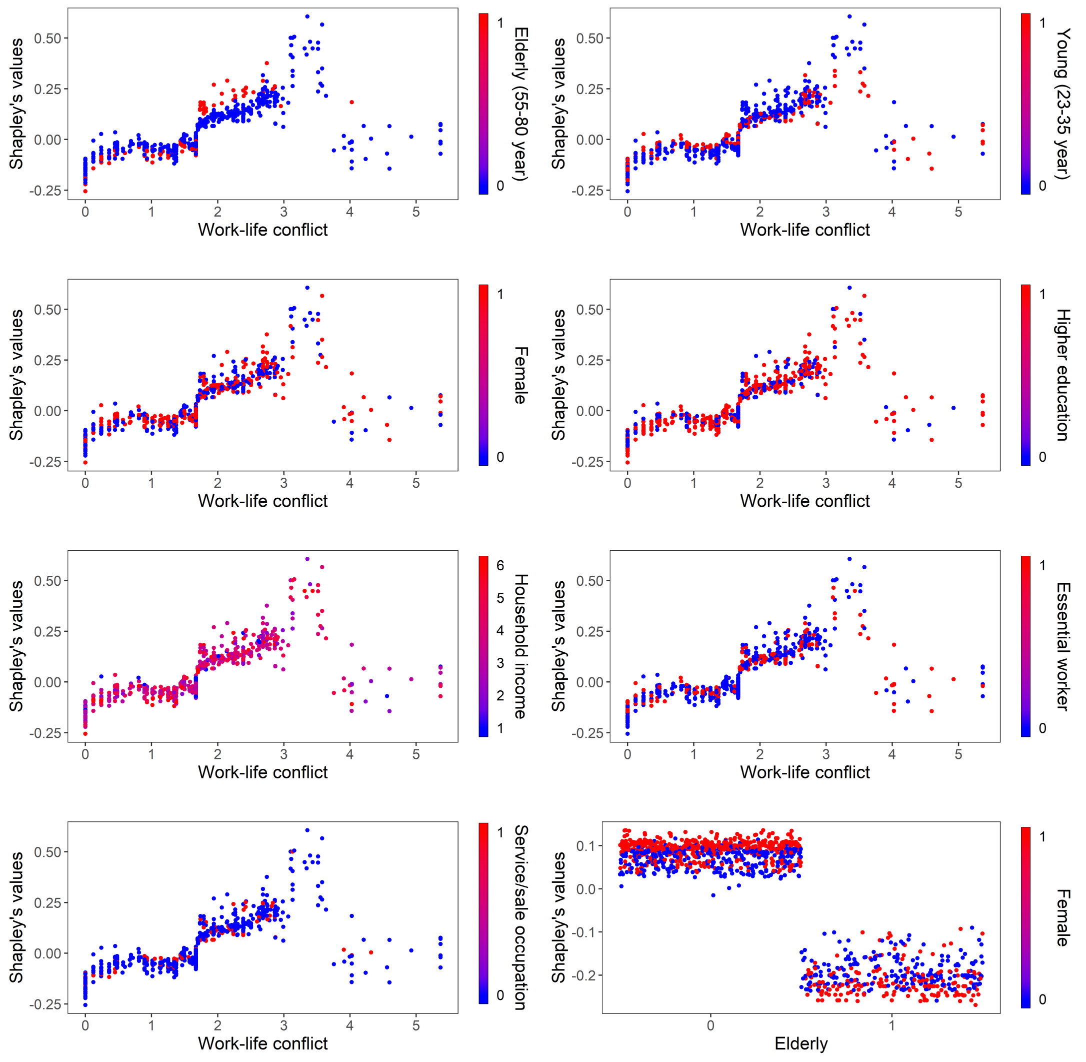

SHAP dependence plot in 2020, interaction between work-life conflict and other features in predicting exposure. The bottom-right plot shows the interaction between older age and gender on model prediction.

G.3. *Interaction between NPI and key features in 2021.*

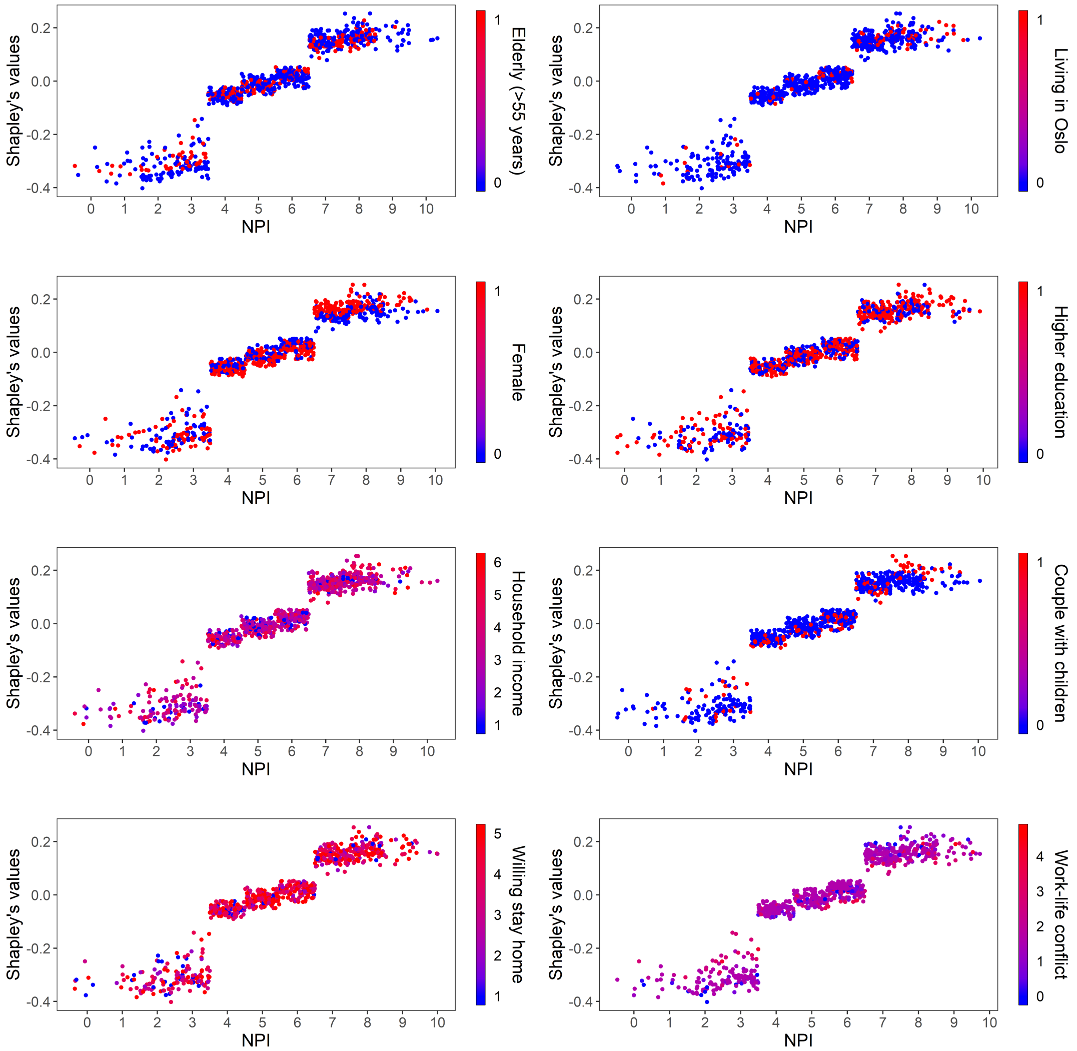

G.4. *Interaction between work-life conflict and key features in 2021.*

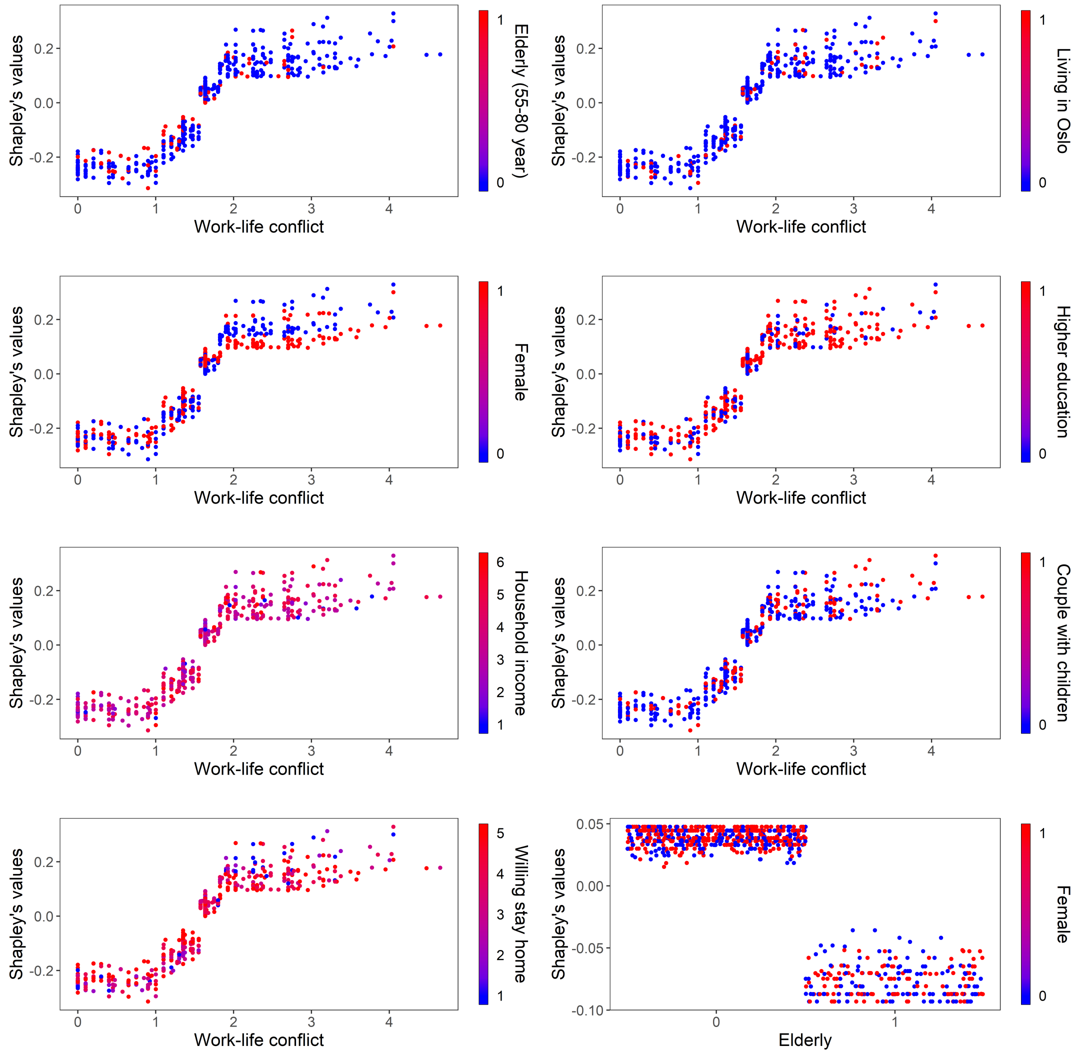

SHAP dependence plot in 2021, interaction between work-life conflict and other features in predicting exposure. The bottom-right plot shows the interaction between older age and gender on model prediction.

References

Bakkeli, N. Z. (2022). Predicting Psychological Distress During the COVID-19 Pandemic: Do Socioeconomic Factors Matter? *Social Science Computer Review*.

Chen, Y.-H., Glymour, M., Riley, A., Balmes, J., Duchowny, K., Harrison, R., Matthay, E., and Bibbins-Domingo, K. (2021). Excess mortality associated with the COVID-19 pandemic among Californians 18–65 years of age, by occupational sector and occupation: March through November 2020. *PLOS ONE*, 16(6):e0252454. Publisher: Public Library of Science.

Harper, C. A., Satchell, L. P., Fido, D., and Latzman, R. D. (2021). Functional Fear Predicts Public Health Compliance in the COVID-19 Pandemic. *International Journal of Mental Health and Addiction*, 19(5):1875–1888.

Hellevik, O. (2016). Extreme nonresponse and response bias. *Quality & Quantity*, 50(5):1969–1991.

Jørgensen, F., Bor, A., and Petersen, M. B. (2021). Compliance without fear: Individual-level protective behaviour during the first wave of the COVID-19 pandemic. *British Journal of Health Psychology*, 26(2):679–696.

Lanciano, T., Graziano, G., Curci, A., Costadura, S., and Monaco, A. (2020). Risk Perceptions and Psychological Effects During the Italian COVID-19 Emergency. *Frontiers in Psychology*, 11.

Li, S., Jiang, L., Li, X., Lin, F., Wang, Y., Li, B., Jiang, T., An, W., Liu, S., Liu, H., Xu, P., Zhao, L., Zhang, L., Mu, J., Wang, H., Kang, J., Li, Y., Huang, L., Zhu, C., Zhao, S., Lu, J., Ji, J., and Zhao, J. (2020). Clinical and pathological investigation of patients with severe COVID-19. *JCI Insight*, 5(12):e138070.

Lu, W., Wang, H., Lin, Y., and Li, L. (2020). Psychological status of medical workforce during the COVID-19 pandemic: A cross-sectional study. *Psychiatry Research*, 288:112936.

Muller, A. E., Hafstad, E. V., Himmels, J. P. W., Smedslund, G., Flottorp, S., Stensland, S. i., Stroobants, S., Van de Velde, S., and Vist, G. E. (2020). The mental health impact of the covid-19 pandemic on healthcare workers, and interventions to help them: A rapid systematic review. *Psychiatry Research*, 293:113441.

Ni, M. Y., Yang, L., Leung, C. M. C., Li, N., Yao, X. I., Wang, Y., Leung, G. M., Cowling, B. J., and Liao, Q. (2020). Mental Health, Risk Factors, and Social Media Use During the COVID-19 Epidemic and Cordon Sanitaire Among the Community and Health Professionals in Wuhan, China: Cross-Sectional Survey. *JMIR Mental Health*, 7(5):e19009.

Niño, M., Harris, C., Drawve, G., and Fitzpatrick, K. M. (2021). Race and ethnicity, gender, and age on perceived threats and fear of COVID-19: Evidence from two national data sources. *SSM - Population Health*, 13:100717.

Pijls, B. G., Jolani, S., Atherley, A., Derckx, R. T., Dijkstra, J. I. R., Franssen, G. H. L., Hendriks, S., Richters, A., Venemans-Jellema, A., Zalpuri, S., and Zeegers, M. P. (2021). Demographic risk factors for COVID-19 infection, severity, ICU admission and death: a meta-analysis of 59 studies. *BMJ Open*, 11(1):e044640.

Prasad, K., McLoughlin, C., Stillman, M., Poplau, S., Goelz, E., Taylor, S., Nankivil, N., Brown, R., Linzer, M., Cappelucci, K., Barbouche, M., and Sinsky, C. A. (2021). Prevalence and correlates of stress and burnout among U.S. healthcare workers during the COVID-19 pandemic: A national cross-sectional survey study. *eClinicalMedicine*, 35.

Proto, E. and Quintana-Domeque, C. (2021). COVID-19 and mental health deterioration by ethnicity and gender in the UK. *PLOS ONE*, 16(1):e0244419.

SSB (2011). Standard Classification of Occupations [Standard for yrkesklassifisering: (STYRK-08)]. Technical Report Notater 17/2011, Statistics Norway, Oslo-Kongsvinger.

Thomas, M. D., Matthay, E. C., Duchowny, K. A., Riley, A. R., Khela, H., Chen, Y.-H., Bibbins-Domingo, K., and Glymour, M. M. (2022). A descriptive analysis of 2020 California Occupational Safety and Health Administration covid-19-related complaints. *SSM - Population Health*, 17:101016.

Wu, X., Li, X., Lu, Y., and Hout, M. (2021). Two tales of one city: Unequal vulnerability and resilience to COVID-19 by socioeconomic status in Wuhan, China. *Research in Social Stratification and Mobility*, 72:100584.
